# Mondo/ChREBP-Mlx-Regulated Transcriptional Network Is Essential for Dietary Sugar Tolerance in *Drosophila*

**DOI:** 10.1371/journal.pgen.1003438

**Published:** 2013-04-04

**Authors:** Essi Havula, Mari Teesalu, Tuulia Hyötyläinen, Heini Seppälä, Kiran Hasygar, Petri Auvinen, Matej Orešič, Thomas Sandmann, Ville Hietakangas

**Affiliations:** 1 Institute of Biotechnology, University of Helsinki, Helsinki, Finland; 2 Department of Biosciences, University of Helsinki, Helsinki, Finland; 3 VTT Technical Research Centre of Finland, Espoo, Finland; 4 German Cancer Research Center (DKFZ), Heidelberg, Germany; University of California San Francisco, United States of America

## Abstract

Sugars are important nutrients for many animals, but are also proposed to contribute to overnutrition-derived metabolic diseases in humans. Understanding the genetic factors governing dietary sugar tolerance therefore has profound biological and medical significance. Paralogous Mondo transcription factors ChREBP and MondoA, with their common binding partner Mlx, are key sensors of intracellular glucose flux in mammals. Here we report analysis of the *in vivo* function of *Drosophila melanogaster* Mlx and its binding partner Mondo (ChREBP) in respect to tolerance to dietary sugars. Larvae lacking *mlx* or having reduced *mondo* expression show strikingly reduced survival on a diet with moderate or high levels of sucrose, glucose, and fructose. *mlx* null mutants display widespread changes in lipid and phospholipid profiles, signs of amino acid catabolism, as well as strongly elevated circulating glucose levels. Systematic loss-of-function analysis of Mlx target genes reveals that circulating glucose levels and dietary sugar tolerance can be genetically uncoupled: Krüppel-like transcription factor Cabut and carbonyl detoxifying enzyme Aldehyde dehydrogenase type III are essential for dietary sugar tolerance, but display no influence on circulating glucose levels. On the other hand, Phosphofructokinase 2, a regulator of the glycolysis pathway, is needed for both dietary sugar tolerance and maintenance of circulating glucose homeostasis. Furthermore, we show evidence that fatty acid synthesis, which is a highly conserved Mondo-Mlx-regulated process, does not promote dietary sugar tolerance. In contrast, survival of larvae with reduced *fatty acid synthase* expression is sugar-dependent. Our data demonstrate that the transcriptional network regulated by Mondo-Mlx is a critical determinant of the healthful dietary spectrum allowing *Drosophila* to exploit sugar-rich nutrient sources.

## Author Summary

Diet displays extreme natural variation between animal species, which range from highly specialized carnivores, herbivores, and nectarivores to flexible dietary generalists. Humans are not identical in this respect either, but the genetic background likely defines the framework for a healthy diet. However, we understand poorly the genetic factors that define the spectrum of healthy diet for a given species or individual. Here we have explored the genetic basis of dietary sugar tolerance of *Drosophila melanogaster*. *D. melanogaster* is a generalist fruit breeder that feeds on micro-organisms on decaying fruits and vegetables with varying sugar content. However, mutants lacking the conserved Mondo-Mlx transcription factor complex display striking intolerance towards dietary sucrose, glucose, or fructose. This is manifested in the larvae by the inability to grow and pupate on sugar-rich food, including red grape, which belongs to the normal diet of wild *D. melanogaster*. Larvae lacking Mondo-Mlx show widespread metabolic imbalance, including highly elevated circulating glucose. Genome-wide gene expression analysis combined with systematic loss-of-function screening of Mlx targets reveal that the genetic network providing sugar tolerance includes a secondary transcriptional effector as well as regulators of glycolysis and detoxification of reactive metabolites.

## Introduction

Mono- and disaccharides, i.e. sugars, are an important source of nutritional energy, but animal species display marked differences in the degree of sugar utilization and tolerance. While the diet of carnivores is typically low in sugars, nectarivores, like hummingbirds, feed primarily on sugar-rich nectar [1], [2]. Sugars from fruits and honey have been part of the ancestral human diet. However, the large quantities of refined sugars consumed by modern humans far exceed those available in natural sources [3]. In fact, it has been proposed that excessive added sugar in the diet, especially fructose, might contribute to the development of metabolic syndrome [3]–[5]. Yet the genetic factors governing the delicate balance between healthful dietary sugar utilization and the sugar overload-induced metabolic disturbance are poorly understood.

*Drosophila* is a well-suited model for exploring the physiological consequences of sugar intake. *Drosophila melanogaster* is a generalist fruit breeder naturally performing well on a broad range of dietary sugars [6]. However, excessive intake of sugars has been shown to cause diabetes-like metabolic changes in *D. melanogaster*, including insulin resistance, elevated circulating glucose and increased adiposity [7], [8]. Dietary sugars have also been shown to shorten *Drosophila* lifespan [9]. The sugar-induced insulin resistance has been attributed to the JNK-regulated lipocalin Neural Lazarillo [8]. Moreover, high sugar induced gene expression has been previously analysed [7], [10]. However, beyond these observations, the functional interactions between genotype and dietary sugar have remained poorly understood.

Elevated systemic glucose levels cause cellular stress and tissue damage [11], [12]. Animals therefore rapidly adapt their metabolism to fluctuating sugar intake, maintaining circulating glucose levels constant. A postprandial increase in circulating glucose triggers the release of insulin, which induces the rapid uptake of excess glucose by metabolic tissues including muscle, adipose tissue, and liver [13]. Intracellular glucose is immediately converted into glucose-6-phosphate and further metabolized into glycogen and lipids or catabolised to release energy. Metabolic tissues are exposed to large variations in the flux of intracellular glucose and therefore need to regulate their metabolism accordingly.

The basic helix-loop-helix transcription factor paralogs ChREBP (Carbohydrate Response Element Binding Protein) and MondoA act together with their common binding partner Mlx (Max-like protein X) to mediate transcriptional responses to intracellular glucose in mammals [14]. The ChREBP/MondoA-Mlx complex is activated by glucose-6-phosphate and other phosphorylated hexoses, and regulates gene expression by binding to target promoters containing a carbohydrate response element (ChoRE) [15]–[19]. ChREBP and MondoA regulate the majority of the global glucose-induced transcriptional responses and many of their target genes encode enzymes in glycolytic and lipogenic pathways [15], [16], [20], [21]. ChREBP and MondoA play differential tissue-specific roles in mammals: ChREBP functions in the liver, adipose tissue and pancreatic beta cells [22]–[26], while MondoA is predominantly expressed in the skeletal muscle [27].

Of the mammalian ChREBP/MondoA-Mlx complex, the role of ChREBP has been studied in a physiological setting using loss-of-function mice. While *ChREBP* is nonessential in terms of survival, the mutant mice display a number of metabolic phenotypes, including elevated plasma glucose and liver glycogen as well as reduced adiposity [25], [28]. *ChREBP^−/−^* mice survive poorly on a diet with high levels of sugars, but the underlying reasons have remained unexplored [25]. ChREBP is known to regulate a range of metabolic genes, including those involved in *de novo* lipogenesis [15], [21]. Which target genes contribute to the various physiological phenotypes and what is the causal interrelationship between the physiological phenotypes, are questions that require powerful genetics and are therefore challenging to address *in vivo* in mammals. Moreover, existence of another Mondo paralog, MondoA, might mask some phenotypes in the *ChREBP^−/−^* mouse. To better understand the physiological roles of the Mondo/ChREBP/-Mlx complex and its target genes, we have explored their role in *Drosophila melanogaster*. The *Drosophila* genome encodes one ortholog for each of ChREBP/MondoA and Mlx, which we call Mondo (alternative identifiers: CG18362, Mlx interactor, ChREBP) and Mlx (alternative identifiers: CG3350, Bigmax), respectively [27], [29], [30].

We have generated *mlx* null mutant flies, which displayed lethality in the late pupal stage. *D. melanogaster* larvae can normally utilize high levels of dietary sugars [6], but loss of Mlx or knockdown of Mondo caused striking intolerance towards sucrose, glucose and fructose. The *mlx* null mutant larvae also displayed extensive metabolic changes, with strongly elevated circulating glucose, signs of amino acid catabolism and altered lipid and phospholipid profiles. Systematic functional analysis of Mlx-regulated genes revealed three genes contributing to dietary sugar tolerance: *cabut*, encoding a Krüppel-like transcription factor, *phosphofructokinase 2*, a regulator of the glycolytic pathway, and *Aldehyde dehydrogenase type III*, which is linked to detoxification of reactive aldehydes.

## Results

### Mondo-Mlx complex is essential for sugar tolerance

**Figure 1:**
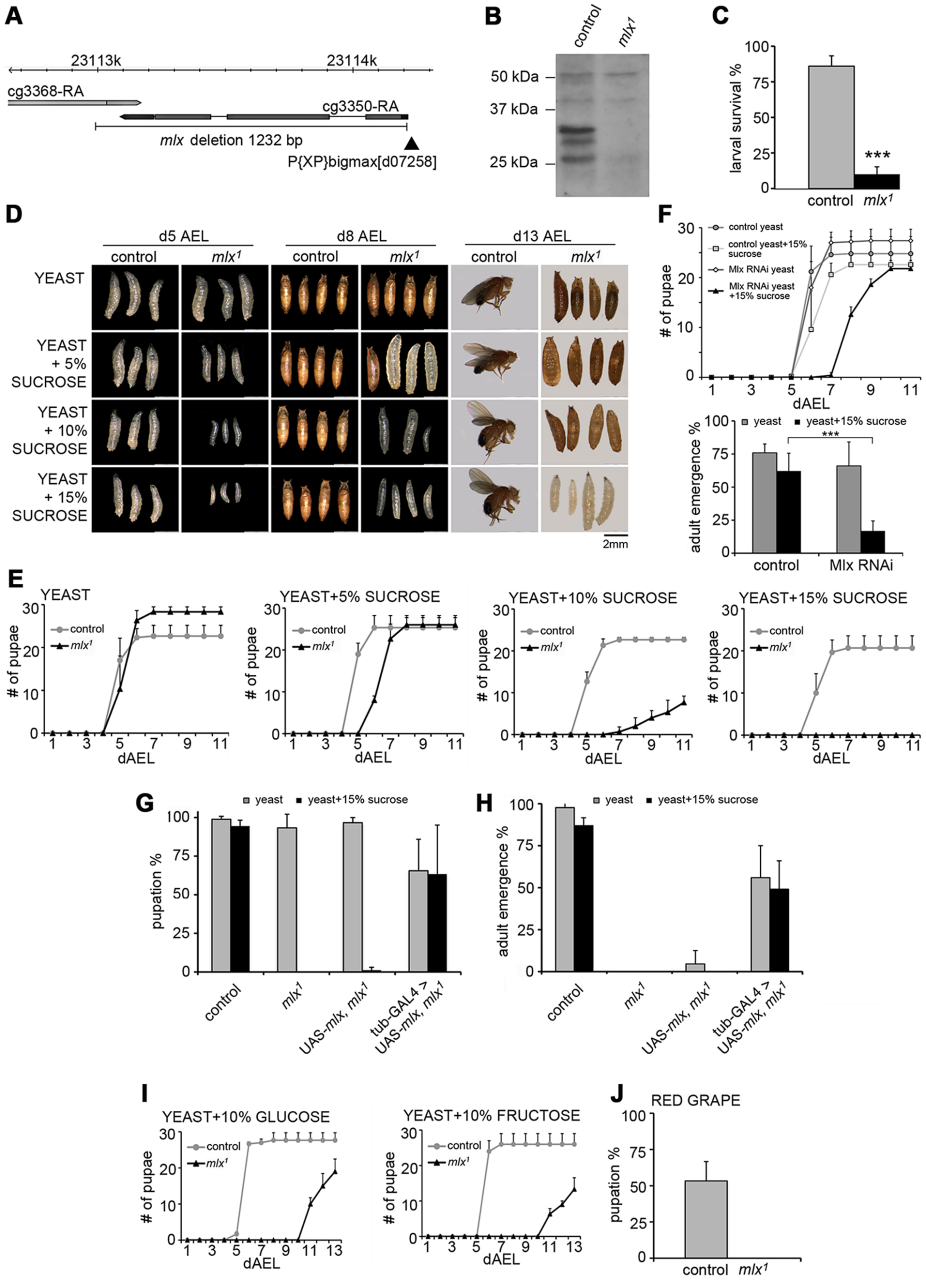
*Drosophila* larvae lacking functional Mlx display strong intolerance to dietary sugars. (A) Schematic presentation of *mlx^1^* mutant locus. P-element is indicated with black arrowhead. (B) Western blot from control and *mlx^1^* mutant larval lysates using anti-Mlx antibodies. (C) Survival of control and *mlx^1^* mutant larvae after 4 days on a 20% sucrose-only diet. (D) Images of control and *mlx^1^* mutants on 20% yeast diet supplemented with 0–15% sucrose after 5, 8, and 13 days after egg laying (dAEL). (E) Pupation kinetics of control and *mlx^1^* mutants on a 20% yeast diet supplemented with 0–15% sucrose. (F) Pupation kinetics and survival of control (tub-GAL4>) and Mlx RNAi (tub-GAL4>Mlx RNAi) larvae on 20% yeast diet with or without 15% sucrose. (G) Rescue of *mlx^1^* mutant pupation on high sugar diet by ubiquitous (tub-GAL4) transgenic expression of *mlx*. (H) Rescue of adult viability of *mlx^1^* mutants by ubiquitous (tub-GAL4) transgenic expression of *mlx*. (I) Pupation kinetics of control and *mlx^1^* mutants on a 20% yeast diet supplemented with 10% glucose or fructose. (J) Pupation % of control and *mlx^1^* mutant larvae on pieces of red grape with baker's yeast inoculum. Error bars represent ± SEM (*** p<0.001).

To study the physiological role of *Drosophila* Mlx, we generated a mutant allele using imprecise P-element (P{XP}*bigmax* ^d07258^) excision. We recovered one mutant allele, *mlx^1^*, which lacked the entire coding region of *mlx* as well as 17 C-terminal amino acids of the neighbouring gene CG3368 (Figure 1A). As controls, we recovered lines from which the P-element had been excised precisely, leaving *mlx* intact. If not differently stated, the precise excision line is used as a control throughout the study. As expected, *mlx^1^* mutant larvae expressed neither Mlx protein nor mRNA (Figure 1B; Figure S1A). The *mlx^1^* mutants displayed lethality at the late pupal (pharate) stage (Figure S1B), and only a small number of adult flies could be recovered. *mlx^1^* mutant flies also displayed a modest developmental delay when raised on our standard fly food (Figure S1C).

Experiments with defined nutrients revealed that *mlx^1^* mutant larvae failed to survive on a diet with 20% sucrose as the sole nutrient source (Figure 1C). To test if the mutant lethality was due to either the inability to utilize carbohydrates as energy source or intolerance towards sucrose, we supplemented protein-rich diet (20% yeast) with increasing levels of sucrose. While *mlx^1^* mutant larvae developed with similar kinetics as control animals on a high protein/low sugar diet, increasing the sucrose concentration gradually slowed down larval development of *mlx^1^* mutants (Figure 1D,E). At higher sucrose levels, *mlx^1^* mutants failed to pupate and died as larvae, while control animals displayed no apparent change in pupation kinetics with respect to 0–15% sucrose.

To confirm that the observed phenotypes were due to loss of *mlx* function, we used RNAi-mediated knockdown and transgenic rescue. Ubiquitous knockdown of Mlx by RNAi led to significantly slower pupation, and increased pupal lethality on protein rich food supplemented with 15% sucrose, while displaying no visible phenotype in the absence of added sucrose (Figure 1F). Driver line without RNAi was used as a control. Moreover, sugar intolerance and pupal lethality of the *mlx^1^* mutants were efficiently rescued by ubiquitous expression of transgenic *mlx* (tub-GAL4>UAS-*mlx*) (Figure 1G, 1H). To further rule out the possibility that sugar intolerance was due to disturbed function of the neighbouring gene CG3368, we used RNAi for ubiquitous knockdown. CG3368 was efficiently silenced, but no sugar intolerance was observed (Figure S1D–S1F). Thus, *mlx* gene function is essential for tolerance to dietary sucrose.

To test for specific intolerance towards glucose or fructose, we supplemented the protein-rich food with 10% of either monosaccharide. Both caused clear developmental delays of *mlx^1^* mutants (Figure 1I). *Drosophila melanogaster* is a dietary generalist, feeding on micro-organisms on decaying fruits and vegetables that have varying sugar content. To test whether the sugar intolerance of *mlx^1^* mutants was relevant within the natural spectrum of *D. melanogaster*'s diet, we allowed larvae to develop on pieces of red grape with baker's yeast inoculum. Indeed, *mlx^1^* mutants were unable to pupate in these conditions, while >50% of the control larvae reached the pupal stage (Figure 1J).

**Figure 2:**
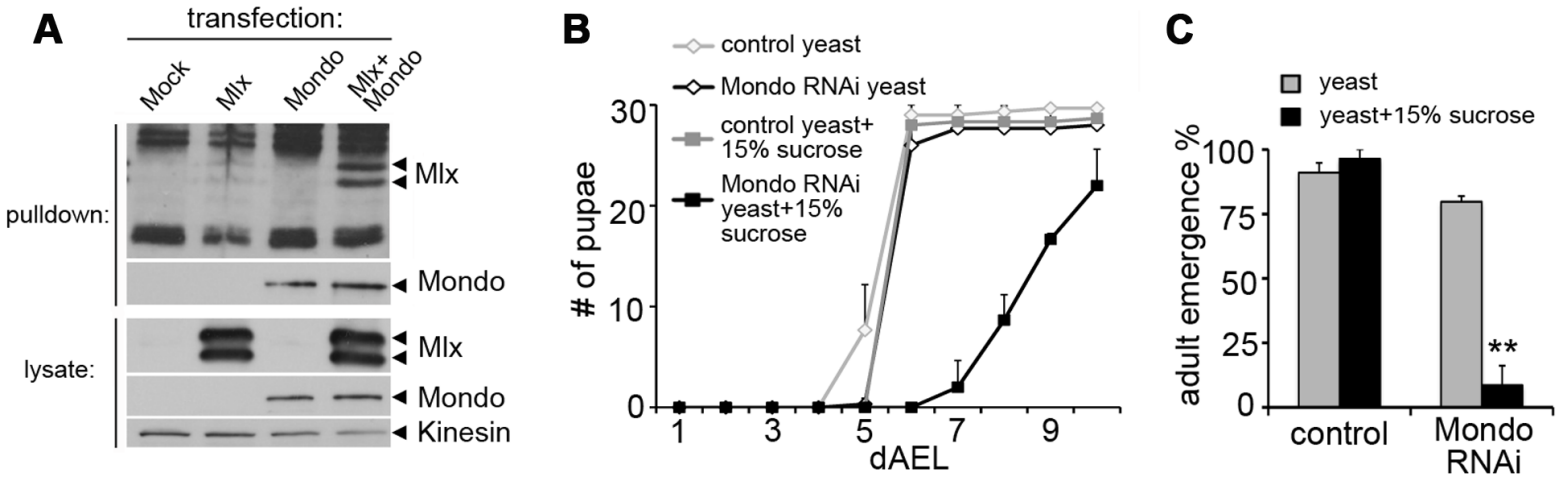
Evidence for functional Mondo-Mlx heterodimer. (A) Co-purification of Mlx upon pulldown of Strep-tagged Mondo from S2 cells. Mlx was detected on Western blot using anti-Mlx antibodies. Mondo-Strep-V5 was detected using anti-V5 antibodies. Kinesin was used as loading control. (B) Pupation kinetics of control (tub-GAL4>) and Mondo RNAi (tub-GAL4>Mondo RNAi) larvae on 20% yeast diet with or without 15% sucrose. (C) Survival into adulthood of control (tub-GAL4>) and Mondo RNAi (tub-GAL4>Mondo RNAi) animals grown on 20% yeast diet with or without 15% sucrose. Error bars represent ± SEM (** p<0.01).

In mammals, Mlx forms a functional complex with the Mondo paralogs, MondoA and ChREBP. We tested if the heterodimeric function of Mlx is conserved in *Drosophila* and essential for the sugar tolerance. *Drosophila* Mlx co-immunoprecipitated with Mondo when expressed in *Drosophila* S2 cells, suggesting heterodimeric function (Figure 2A). Notably, Mlx from S2 cells runs as two distinct bands (Figure 2A), which correspond to the two upper bands present in the *in vivo* sample (Figure 1B and Figure S2). The nature of these bands has remained unclear, no alternative splicing has been reported and both bands are resistant to alkaline phosphatase treatment (not shown). Ubiquitous RNAi knockdown of Mondo (tub-GAL4>Mondo RNAi) led to delayed pupation (Figure 2B) and reduced pupal survival on high sugar diet (20% yeast-15% sucrose) (Figure 2C). In sum, the biochemical and genetic evidence implies conservation of the Mondo-Mlx heterodimer function in *Drosophila*.

### Changes in lipid, amino acid, and carbohydrate metabolism in *mlx* mutants

**Figure 3:**
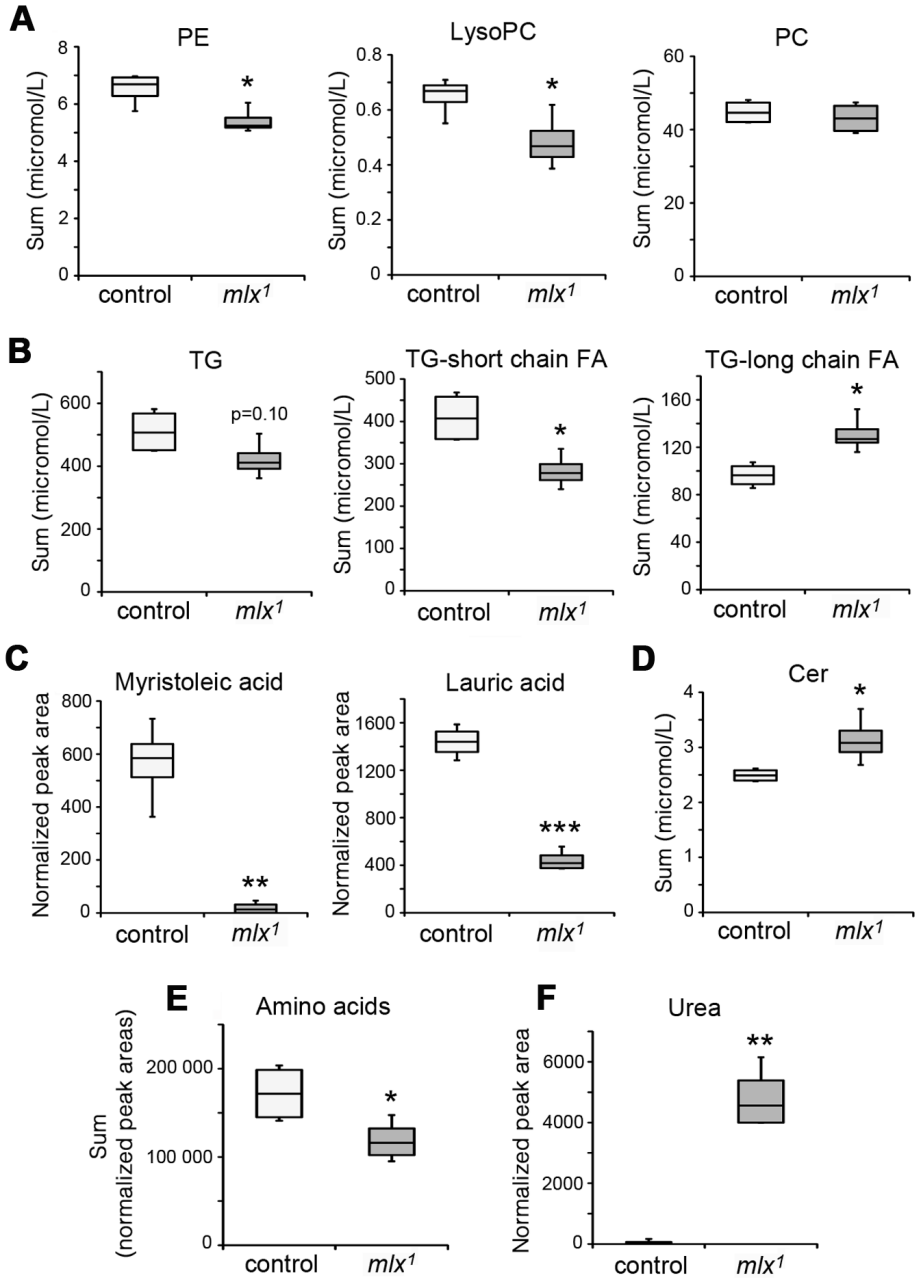
Changes in lipid and amino acid profiles in *mlx^1^* mutants. (A) Phospholipid levels in control and *mlx^1^* mutant larvae. PE: phosphatidylethanolamine; LysoPC: lysophosphatidylcholine; PC: phosphatidylcholine. (B) Total triglyceride levels as well as triglycerides divided into two groups based on total fatty acid length. For TG-long chain FA group, total fatty acid carbon number >48. (C) Levels of myristoleic acid and lauric acid were strongly reduced in *mlx^1^* mutant larvae. (D) Ceramide levels in control and *mlx^1^* mutant larvae. (E) Total levels of amino acids in control and *mlx^1^* mutant larvae. (F) Urea levels in control and *mlx^1^* mutant larvae. Error bars represent ± SEM (*p<0.05; ** p<0.01; *** p<0.001). The box contains the middle 50% of the data, the two ends of the boxes represent the upper and the lower quartile of the data. The median is represented by the line in the center of the rectangular box. The vertical lines mark the minimum and maximum data values.

The diet-dependent phenotype of *mlx^1^* mutants led us to perform a comprehensive survey of their metabolic status using mass-spectrometry based lipidomics and metabolomics. Lipidomics analysis revealed significant downregulation of key phospholipid groups, such as phosphatidylethanolamines (PE) and lysophosphatidylcholines (LysoPC) (Figure 3A). Total triglyceride (TG) levels showed a lower trend in *mlx^1^* mutants, but the difference to the controls was not statistically significant (Figure 3B). However, *mlx^1^* mutants showed significant enrichment in triglyceride species with long fatty acid tails (Figure 3B). At the same time, *mlx^1^* mutants showed strong downregulation of certain fatty acids, such as myristoleic acid and lauric acid (Figure 3C). On the other hand, ceramide (Cer) levels were elevated in *mlx^1^* mutants compared to controls (Figure 3D). Together, *mlx^1^* mutants display signs of severely altered lipid and phospholipid metabolism. In addition to the changes in lipid profiles, total amino acid levels were significantly reduced in *mlx^1^* mutants (Figure 3E), while concentration of urea was dramatically increased (Figure 3F). This implies that *mlx^1^* mutants might catabolise amino acids for energy.

**Figure 4:**
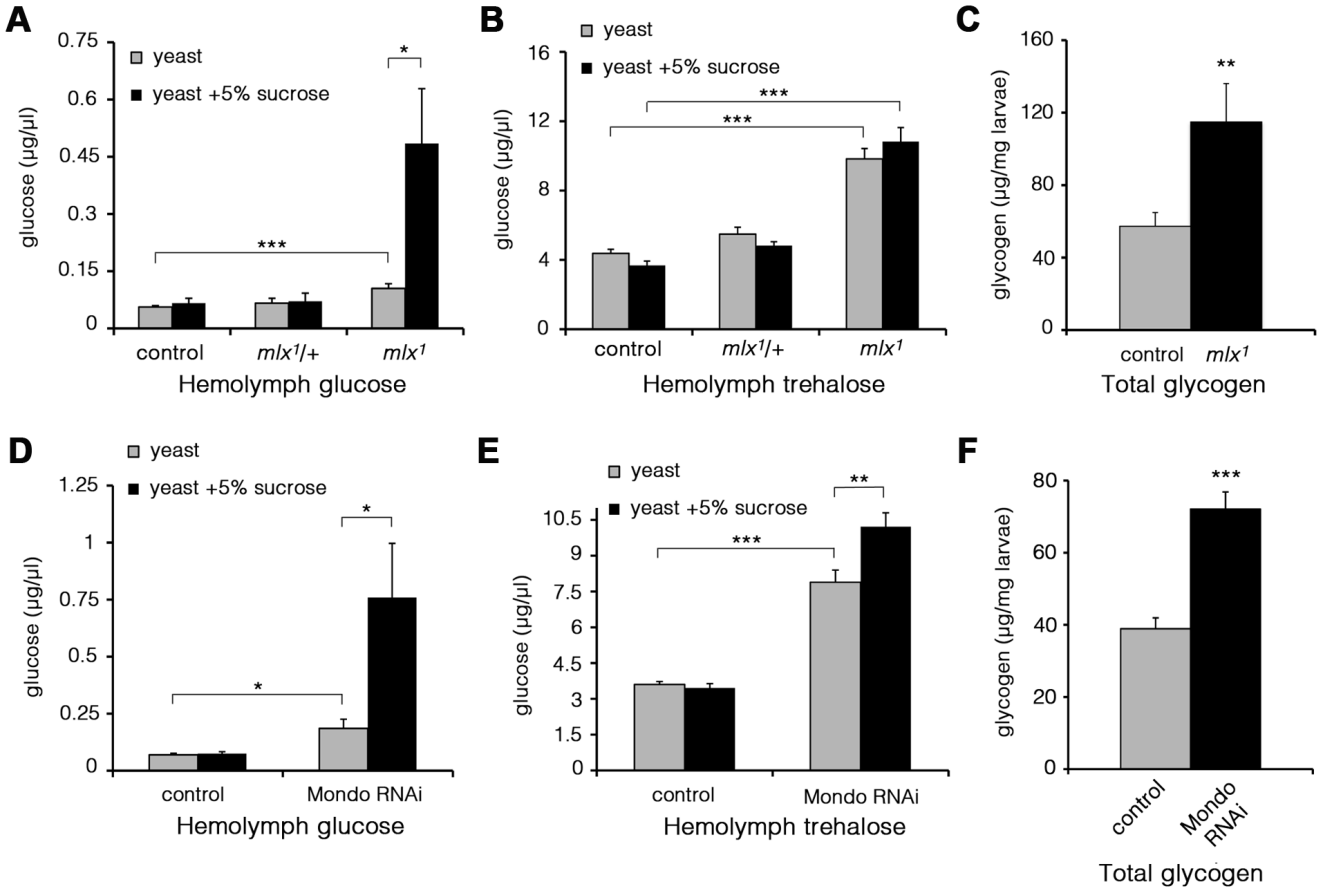
Elevated glucose, trehalose, and glycogen levels in the absence of Mondo-Mlx. Hemolymph glucose (A) and trehalose (B) levels in control and *mlx^1^* mutant larvae on a 20% yeast diet with or without 5% sucrose. (C) Glycogen levels in control and *mlx^1^* mutant larvae on a 20% yeast-5% sucrose diet. Hemolymph glucose (D) and trehalose (E) levels in control (tub-GAL4>) and Mondo RNAi (tub-GAL4>Mondo RNAi) larvae grown on a 20% yeast-5% sucrose diet. (F) Glycogen levels in control (tub-GAL4>) and Mondo RNAi (tub-GAL4>Mondo RNAi) larvae on 20% yeast-5% sucrose diet. Error bars represent ± SEM (* p<0.05; ** p<0.01; *** p<0.001).

To study changes in glucose metabolism, we measured glucose and trehalose levels in the hemolymph of larvae raised on diets with varying sucrose content. Trehalose is a disaccharide released by gluconeogenesis and glycogenolysis in insects [31]. The levels of circulating glucose were moderately elevated in *mlx^1^* mutant larvae raised on a low-sugar diet (20% yeast) (Figure 4A). However, increasing the dietary sucrose to 5%, which still sustains larval development of *mlx^1^* mutants, led to a prominent increase of circulating glucose in *mlx^1^* mutants while remaining constant in control animals (Figure 4A). Trehalose levels were also significantly elevated in *mlx^1^* mutants, but unlike glucose, trehalose levels were little affected by the dietary sucrose levels (Figure 4B). Furthermore, glycogen levels were significantly elevated in *mlx^1^* mutants (Figure 4C), indicating that glucose catabolism, not cellular glucose intake, is limiting glucose clearance from circulation in *mlx^1^* mutants.

To verify that the elevated glucose levels were due to loss of Mlx function, we performed a transgenic rescue, which normalized circulating glucose levels (Figure S3A). Further, RNAi-mediated knockdown of Mlx led to a clear increase in circulating glucose, trehalose and glycogen (Figure S3B–S3D). In line with the expectation of heterodimeric function for Mondo and Mlx proteins, Mondo RNAi knockdown led to a prominent increase in circulating glucose and trehalose (Figure 4D,E). Also the glycogen levels were significantly increased in Mondo RNAi larvae (Figure 4F). Knockdown of CG3368, the neighbouring gene of *mlx*, had no influence on circulating glucose or trehalose (Figure S3B, S3C). In conclusion, *mlx* null mutant and *mondo* knockdown larvae display a reduced capacity to utilize circulating glucose leading to poor homeostatic adaptation to elevated dietary sugar.

### Mlx is functionally important in the fat body

**Figure 5:**
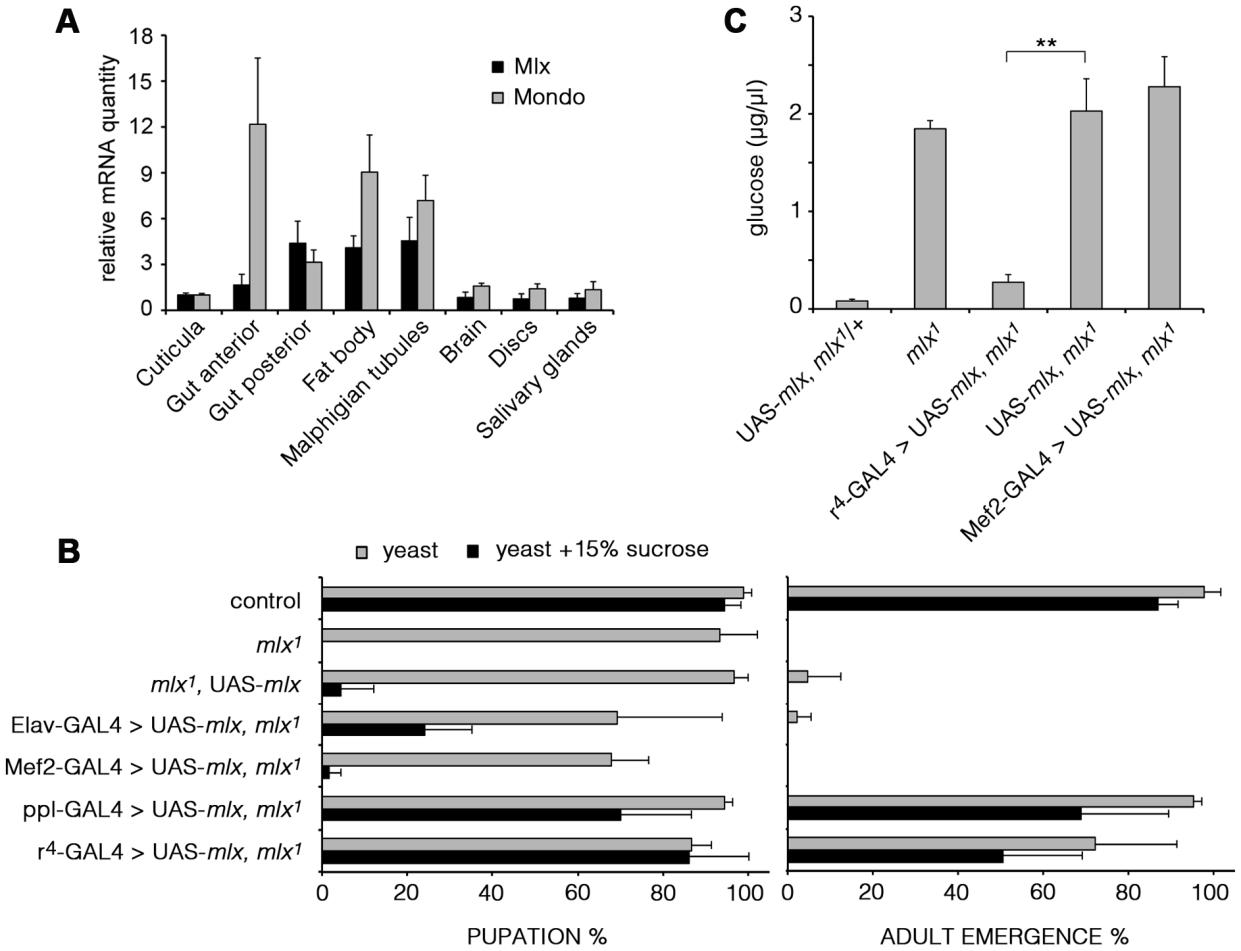
Mlx is functionally important in the fat body. (A) mRNA expression of *mondo* and *mlx* in dissected tissues of 3^rd^ instar larvae measured by quantitative RT-PCR. *rp49* was used as a reference gene. (B) Tissue specific rescue of pupation and adult emergence of the *mlx^1^* mutants using neuronal (Elav), muscle (Mef2), or fat body (ppl and r^4^) –specific GAL4 drivers. (C) Fat body –specific (r^4^-GAL4) expression of transgenic *mlx* rescues the elevated circulating glucose in *mlx^1^* mutants. Error bars represent ± SEM (** p<0.01).

As mammalian MondoA-Mlx and ChREBP-Mlx appear to display tissue-specific functions, we wanted to determine the functionally important tissues for *Drosophila* Mondo-Mlx. We first analyzed their mRNA expression using quantitative RT-PCR. Expression of Mondo and Mlx were highly correlated; highest levels were detected in the fat body, gut and Malpighian tubules (Figure 5A). To functionally dissect the contribution of different tissues to the sugar sensitivity, we used tissue-specific transgenic rescue. Restoring Mlx expression in neurons (Elav-GAL4) or muscle (Mef2-GAL4) did not significantly improve the sugar tolerance or survival of *mlx^1^* mutants (Figure 5B). However, targeted expression in the fat body with two independent driver lines (ppl- and r^4^-GAL4) efficiently rescued survival on high sugar diet. Moreover, rescue of Mlx in the fat body, but not in muscle, was sufficient to normalize the levels of circulating glucose in *mlx^1^* mutants (Figure 5C). Notably, transgenic Mlx expression by tub-GAL4 (Figure 1G) or Mef2-GAL4 (Figure 5B) caused moderately reduced survival, which was also observed in the presence of intact endogenous *mlx* (Figure S4).

### Mlx regulates metabolic genes and a secondary transcriptional effector

**Figure 6:**
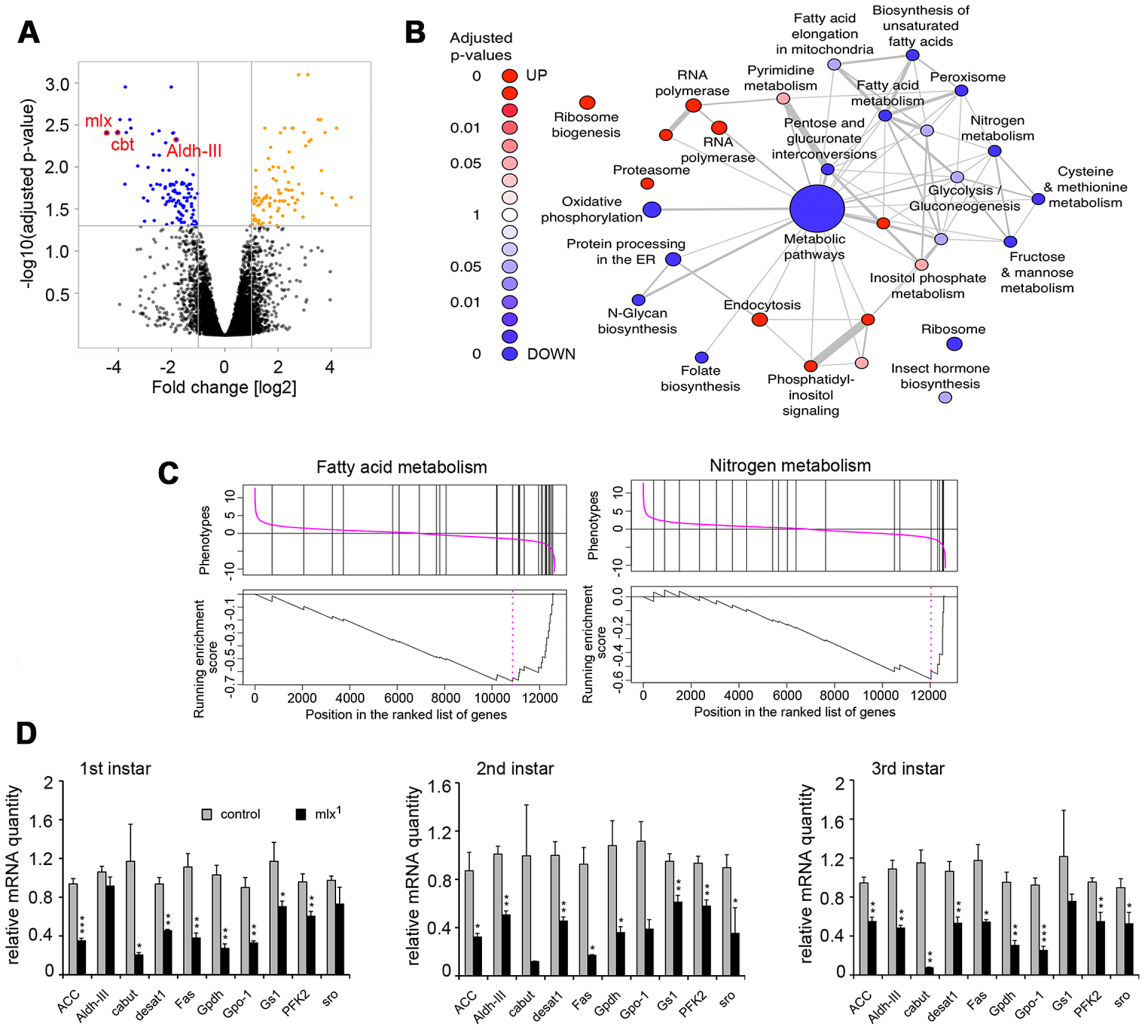
Mlx-regulated gene expression program. (A) Volcano plot indicating genes differentially expressed between control and *mlx^1^* mutant fat bodies. Significant changes (p<0.05 and log2 fold changes>±1) are indicated in orange (upregulated in *mlx^1^*) and blue (downregulated in *mlx^1^*). (B) Results of a gene-set enrichment analysis (GSEA) of KEGG pathways. Red: upregulated set in *mlx^1^* mutant, blue: downregulated set. The size of nodes illustrates the size of gene sets and the width of edges denotes the overlap between two gene sets. (C) GSEA charts for KEGG categories “Fatty acid metabolism” and “Nitrogen metabolism”. (D) Developmental stage-specific mRNA expression (quantitative-RT-PCR) of selected genes downregulated in *mlx^1^* mutant in the microarray. Expression levels were normalized using *Actin 42A* as a reference gene. Error bars represent ± SEM (* p<0.05; ** p<0.01; *** p<0.001).

To identify Mlx target genes, we performed a microarray gene expression profiling specifically in the fat body. Comparing gene expression between *mlx^1^* mutant and control fat bodies from 3^rd^ instar prewandering larvae raised on a moderate sucrose level diet (20% yeast-5% sucrose) revealed 97 down- and 96 up-regulated genes (>2-fold change and adjusted p-value<0.05) (Figure 6A; Table S1). As expected for a deletion mutant, *mlx* was identified as the most strongly downregulated gene on the microarray (Figure 6A). Gene Set Enrichment Analysis (GSEA) revealed a significant enrichment of KEGG categories involved in metabolic regulation (Figure 6B). For example, KEGG categories of fatty acid metabolism and nitrogen metabolism were strongly downregulated (Figure 6C), which is in good agreement with the metabolomics data (Figure 3). Many of the genes downregulated in *mlx^1^* mutant fat body also showed reduced expression during earlier larval stages in whole larval samples (Figure 6D). Mlx-regulated genes include several key metabolic genes, such as *glycerol-3-phosphate dehydrogenase-1* (*Gpdh*, CG9042), *stearoyl-CoA 9-desaturase-1* (*desat1*, CG5887), *Glutamine synthetase-1* (*Gs1*, CG2718), and *3-hydroxybutyrate dehydrogenase* (*sro*, CG12068). The mean expression levels of three known ChREBP and MondoA targets in mammals, *fatty acid synthase* (*Fas*, CG3523), *acetyl-CoA carboxylase* (*ACC*, CG11198) and *phosphofructokinase 2* (*PFK2*, 6-phosphofructo-2-kinase/fructose-2,6-bisphosphatase, CG3400), [20], [32], were also reduced, although they did not pass our strict microarray cut-offs (Figure 6D). Interestingly, one of the most strongly downregulated genes in *mlx^1^* mutant fat body was that encoding the Krüppel-like transcription factor Cabut (cbt; CG4427) (Figure 6A, 6D).

### Evidence for a transcriptional network mediating sugar tolerance

**Figure 7:**
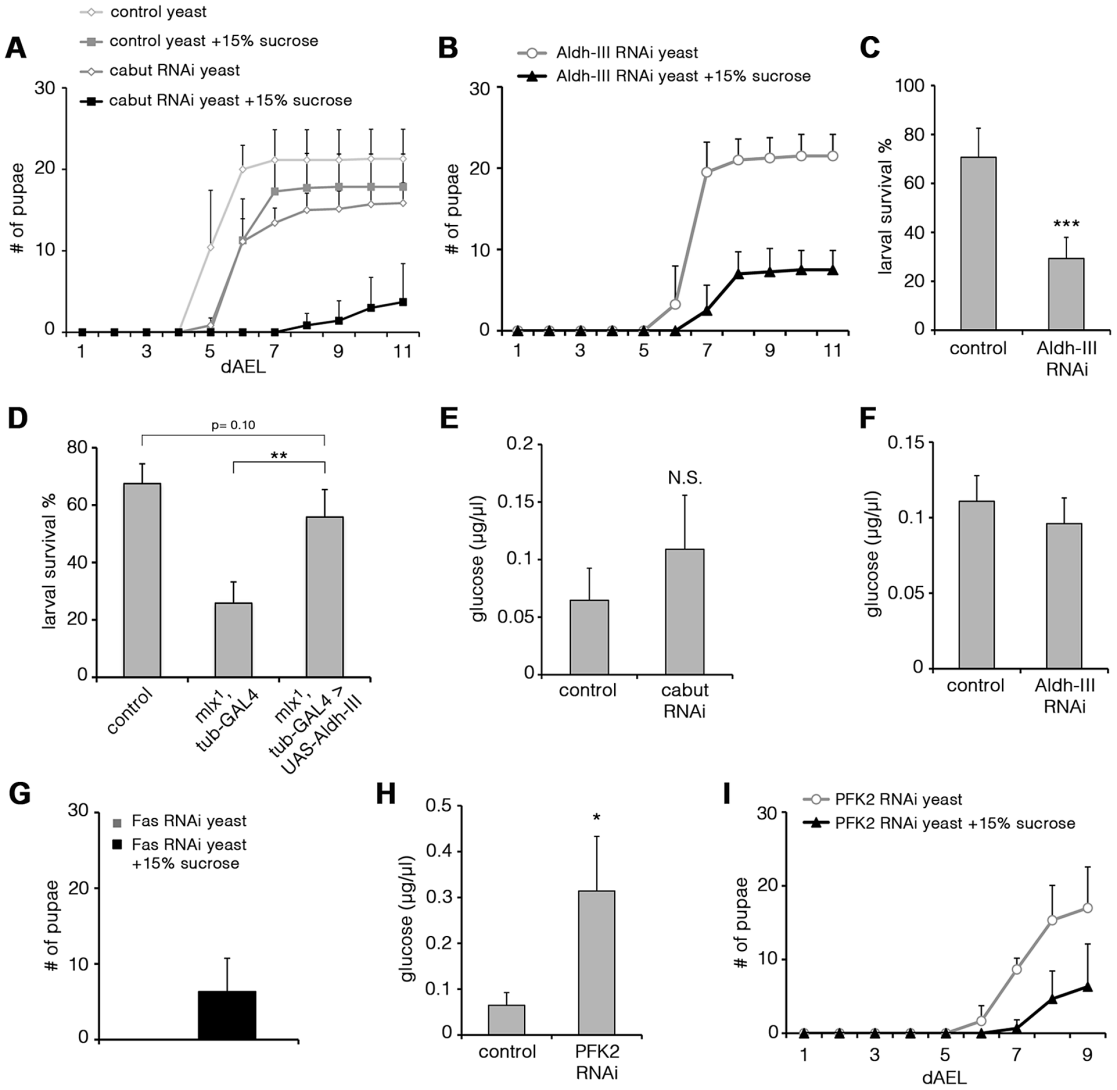
Functional analysis of Mlx-regulated genes. (A) Pupation kinetics of control (tub^ts^-GAL4>) and Cabut RNAi (tub^ts^-GAL4>Cabut RNAi) larvae on 20% yeast diet with or without 15% sucrose. To avoid early larval lethality, we used temperature sensitive GAL80 with tub-GAL4 system to induce RNAi expression during larval stage. Larvae were maintained in +29°C after collection. Cabut knockdown causes a moderate delay also in the low sugar (20% yeast) diet, but addition of 15% substantially reduces survival and delays pupation further. (B) Pupation kinetics of Aldh-III RNAi (tub-GAL4>Aldh-III RNAi) larvae on 20% yeast diet with or without 15% sucrose. (C) Survival of control (tub-GAL4>) and Aldh-III RNAi (tub-GAL4>Aldh-III RNAi) larvae after 6 days on 20% sucrose-only diet. (D) Survival of control, *mlx^1^*, tub-GAL4 and *mlx^1^*, tub-GAL4>UAS-Aldh-III larvae after 4 days on a 20% sucrose-only diet. (E) Hemolymph glucose levels in control (tub^ts^-GAL4>) and Cabut RNAi (tub^ts^-GAL4>Cabut RNAi) larvae on 20% yeast-5% sucrose. (F) Hemolymph glucose levels in control (tub-GAL4>) and Aldh-III RNAi (tub-GAL4>Aldh-III RNAi) larvae on 20% yeast-5% sucrose. (G) Total pupation of Fas RNAi (tub-GAL4>Fas RNAi) larvae on 20% yeast diet with or without 15% sucrose. (H) Hemolymph glucose levels in control (tub-GAL4>) and PFK2 RNAi (tub-GAL4>PFK2 RNAi) larvae on 20% yeast-5% sucrose. (I) Pupation kinetics of PFK2 RNAi (tub-GAL4>PFK2 RNAi) larvae on 20% yeast diet with or without 15% sucrose. Error bars represent ± SEM (* p<0.05; ** p<0.01; *** p<0.001).

To test in an unbiased way if any of the genes downregulated in *mlx^1^* mutants had an essential role in maintaining organismal sugar tolerance, we systematically targeted 103 candidate genes by RNAi (Table S2). Intriguingly, ubiquitous knockdown of two Mlx-regulated genes identified on the microarray led to significant sugar intolerance. Transcription factor Cabut was among the most highly Mlx-regulated genes in the microarray. Ubiquitous knockdown of Cabut expression by RNAi during the larval stage caused a modest delay of pupation on low sugar diet. However, on high sugar diet (20% yeast-15% sucrose) Cabut knockdown led to prominent developmental delay and impaired survival (Figure 7A). This suggests that Mondo-Mlx activates a hierarchical transcriptional network to regulate dietary sugar tolerance with Cabut as an essential downstream effector.

### Uncoupling of dietary sugar tolerance and circulating glucose

Another Mlx-regulated gene, which caused sugar intolerance upon ubiquitous knockdown, was *Aldehyde dehydrogenase type III* (*Aldh-III*, CG11140; Figure 7B). Most Aldh-III knockdown animals reached the pharate stage on a low sugar diet, but died during early pupal stages on a high-sugar diet (Figure S5A). Similar early pupal lethality was observed in *mlx^1^* mutants on sugar concentrations that allowed pupation (data not shown). Survival on a 20% sucrose-only diet was also significantly reduced upon Aldh-III knockdown (Figure 7C). We also tested whether restoring Aldh-III activity by transgenic expression would be sufficient to rescue impaired *mlx^1^* mutant survival on high sugar diet. While transgenic expression of Aldh-III did not rescue *mlx^1^* mutant pupation on 20% yeast-15% sucrose diet (data not shown), larval survival of *mlx^1^* mutants on 20% sucrose-only diet was significantly improved by transgenic *Aldh-III* (Figure 7D). The same was true when Aldh-III expression was rescued only in the fat body (Figure S5B). In conclusion, Aldh-III is essential and sufficient for providing dietary sugar tolerance.

We also explored whether the sugar intolerance observed upon knockdown of Cabut and Aldh-III was associated with elevated circulating glucose levels. Surprisingly, knockdown of either gene did not result in a significant increase in circulating glucose (Figure 7E, 7F). This implies that impaired clearance of circulating glucose is not an essential prerequisite for intolerance to dietary sugars. Instead, these two Mondo-Mlx-regulated parameters can be uncoupled at the level of the downstream target genes. Based on these phenotypes, the Cabut-dependent branch of the transcriptional network mediates only a subset of Mondo-Mlx functions. We also studied the possibility that Cabut could be a direct regulator of Aldh-III, however the mRNA levels of Aldh-III were unchanged in Cabut RNAi larvae (Figure S5C). Also the mRNA levels of Mondo and Mlx were unchanced in Cabut RNAi larvae, indicating that Cabut is not a feedback regulator of Mondo-Mlx. Notably, it is possible that Cabut is regulating a subset of common genes with Mondo-Mlx.

### Interference of fatty acid synthesis makes larvae dependent on sugar-rich diet

Perhaps the best established function of mammalian ChREBP-Mlx is promotion of *de novo* lipogenesis in response to high carbohydrate intake [14]. This function is mediated through upregulation of lipogenic genes and it appears to be conserved in *Drosophila* (Figure 6D; [33]). Two key targets of Mondo-Mlx involved in *de novo* lipogenesis are *acetyl-CoA carboxylase* (*ACC*) and *fatty acid synthase* (*Fas*). Thus exploring their function in respect to dietary sugar will reveal whether *de novo* lipogenesis is functionally coupled to sugar tolerance. While knockdown of ACC was embryonic lethal (data not shown), Fas knockdown animals displayed some degree of survival until pupal stage. Strikingly, Fas knockdown larvae displayed early larval lethality on high protein diet (20% yeast paste), but diet supplementation with 15% sucrose partially rescued the lethality allowing pupation (Figure 7G). Thus, Mondo-Mlx-mediated regulation of fatty acid synthesis is a non-essential function for dietary sugar tolerance. In contrast, high dietary sugars promote survival upon compromised fatty acid synthesis.

### Phosphofructokinase 2 contributes to circulating glucose and sugar tolerance

As our systematic analysis of sugar tolerance genes did not reveal a mechanism by which Mondo-Mlx maintains low circulating glucose, we analyzed glucose levels on additional Mondo-Mlx targets that have a metabolic function. Phosphofructokinase 2 (PFK2, Pfrx, CG3400) synthesizes and breaks down fructose-2,6-bisphosphate, which is an allosteric activator of phosphofructokinase 1 and hence promotes glycolysis. *PFK2* expression was downregulated in *mlx^1^* mutants (Figure 6D). Knockdown of PFK2 led to elevated circulating glucose, showing that PFK2 is a Mondo-Mlx downstream target, which contributes to circulating glucose levels (Figure 7H). Interestingly, PFK2 knockdown also reduced pupation on high sugar diet (20% yeast-15% sucrose) (Figure 7I), implying that Mondo-Mlx-mediated activation of the glycolytic pathway contributes to dietary sugar tolerance.

## Discussion

Our study demonstrates that interfering with the function of the Mondo-Mlx complex severely affects *Drosophila* energy metabolism, rendering animals highly intolerant to sugars in their diet. The sugar intolerance is likely due to a combined effect of several downstream effectors, since our systematic loss-of-function analysis revealed three Mlx target genes that are essential for survival on high sugar diet. Sugar tolerance is influenced by glycolysis, which also contributes to clearance of glucose from circulation. However, circulating glucose levels and sugar tolerance are phenotypes that can be uncoupled, as in the case of two Mlx targets, Cabut and Aldh-III, which only contribute to sugar tolerance.

Mondo-Mlx shows a high degree of functional conservation between flies and mammals, as orthologs of many *Drosophila* Mlx-regulated genes are known targets of ChREBP/MondoA-Mlx in mammals. These include *glycerol-3-phosphate dehydrogenase-1*, *stearoyl-CoA 9-desaturase-1, fatty acid synthase, acetyl-CoA carboxylase, and phosphofructokinase 2* [15], [21], [25]. *Drosophila* Mlx displayed an essential role in the fat body, which is the counterpart of mammalian adipose tissue and liver. Thus, it is conceivable that the liver and adipose tissue-specific ChREBP-Mlx, instead of the muscle-specific MondoA-Mlx, represents the ancestral function of the heterodimer.

This study identifies *Aldh-III* as a novel gene contributing to dietary sugar tolerance. *Aldh-III* is the ortholog of mammalian *Aldh3* family. Aldehyde dehydrogenases (Aldhs) are highly conserved NAD(P)^+^ -dependent enzymes that oxidize aldehydes to the corresponding carboxylic acid and act on a broad range of substrates [34]. Aldehydes are highly reactive compounds forming adducts with nucleic acids and proteins, thus disturbing cellular functions. Reactive aldehydes can originate from exogenous sources or be products of cellular metabolism. Aldhs have been shown to provide protection against a number of ectopic stresses, including toxic chemicals, heat stress, and UV irradiation [34]–[37]. One of the best-established functions of Aldh proteins is neutralization of acetaldehyde, a toxic metabolite of ethanol. Acetaldehyde elimination is mainly mediated by Aldh2 [34], [38]. Polymorphism in the *Aldh2* gene is common is Asian populations and it leads to poor ethanol tolerance [39]. Our finding that another member of the Aldh family, Aldh-III, is essential for dietary sugar tolerance is intriguing, since there are multiple parallels between the hepatic pathophysiologies related to excessive ethanol and fructose consumption in humans [3]. Of note, we observed that Aldh-III is sufficient in rescuing the sugar intolerance of *mlx^1^* mutants in the fat body, the insect counterpart of liver, suggesting that metabolic stress-induced dysfunction of the fat body contributes to sugar intolerance. Future studies should be aimed at understanding the generation of sugar-derived reactive aldehydes and their molecular targets.

We also provide evidence that *PFK2* expression is positively regulated by Mondo-Mlx in *Drosophila* and that the PFK2 levels are critical in managing circulating glucose levels and providing sugar tolerance. Thus, our data suggests that regulation of *PFK2* gene expression might be a suitable strategy to manage hyperglycemia in diabetes. Elevated *PFK2* expression has also been associated with the high rate of glycolytic flux in neoplastic tumors [40]. Exploring the contribution of Mondo and Mlx proteins in this setting is therefore warranted. In addition to being regulated transcriptionally, PFK2 activity in mammals is known to be posttranslationally regulated by insulin signalling through protein kinase AKT [41], [42]. It will be interesting to learn, what is the contribution of insulin signalling pathway activity on the dietary sugar tolerance.

The finding that Cabut is an essential secondary effector of Mondo-Mlx is intriguing. In fact, *cabut* expression has been reported to respond to other metabolism-related signals: it is upregulated upon inhibition of TOR complex 1 signalling [43], which is likely mediated by activation of the Forkhead transcription factor, FoxA [44]. While the developmental role of Cabut has been studied [45], [46], its metabolic functions have remained unexplored. Our finding showing that Cabut plays an essential metabolic role in providing dietary sugar tolerance implies this topic deserves an in-depth survey in the future. Notably, the closest mammalian homologs of Cabut, Klf-10 and Klf-11, have been linked to metabolic regulation. Mutations in the *klf-11* locus are associated with risk of diabetes [47], while Klf-10 appears to negatively regulate lipogenic genes in hepatocytes [48]. *klf-10* expression is regulated both by circadian signals [49] as well as by ChREBP upon high glucose [48]. Thus, Klf-10 might be the functional ortholog of Cabut. Future studies should be aimed at identifying the Cabut target genes involved in its metabolic functions.

Dietary sugar tolerance displays a wide natural variation, even in closely related animal species. For example, two *Drosophila* species, *D. melanogaster* and *D. mojavensis*, have strikingly different tolerance to dietary sugars [6]. In contrast to the fruit generalist *D. melanogaster*, *D. mojavensis* is a cactus breeder, which does not naturally encounter high levels of simple sugars and displays poor survival on sugar-rich diet [6]. Based on our data, it is possible to hypothesize that Mondo-Mlx-regulated transcriptional network contributes to the natural variation in sugar tolerance. Genetic differences in sugar tolerance are also observed in humans. For example, hereditary fructose intolerance (HFI) is caused by mutations in the *aldolase-B* gene [50]. A fructose-restricted diet renders HFI relatively benign, but ingestion of fructose or sucrose leads to strong symptoms, including nausea and vomiting as well as a risk of liver and kidney damage. It will be important to explore whether genetic changes in the ChREBP/MondoA-Mlx network influence individual's risk for sugar overload-induced metabolic disturbance.

This study highlights the usefulness of *Drosophila* as a model for systematically exploring the genetic factors defining the range of healthful nutrient intake. Notably, the sugar intolerance in *mlx^1^* mutants is not a pleiotropic consequence of a generally disturbed energy metabolism. Knockdown of other key transcriptional metabolic regulators, such as SREBP, did not cause notable sugar intolerance (unpublished observation). The availability of genome-scale reagents, including *in vivo* RNAi lines, offers the possibility for the systematic dissection of genes contributing to dietary sugar tolerance. These genes might include novel members of the Mondo-Mlx-regulated genetic network, but they will also uncover whether parallel regulatory pathways are involved. The *Drosophila* model is also particularly useful in dissecting the function of transcription factors that are master regulators of several gene groups that contribute to distinct physiological outputs. While this study has focused on uncovering those Mondo-Mlx targets that contribute to sugar tolerance and circulating glucose levels, uncovering the downstream effectors behind the other metabolic phenotypes of *mlx^1^* mutant fly awaits for future studies.

## Materials and Methods

### Fly stocks

P{XP}*bigmax* ^d07258^ were obtained from Bloomington stock center. For generating UAS-*mlx* flies, the coding region of *mlx* cDNA was amplified by PCR and cloned into pUAST vector using BglII and XbaI restriction sites. FLAG-tag was incorporated into the C-terminus. Aldh-III coding region was cloned into pUAST vector using NotI and XhoI restriction sites. RNAi lines were obtained from Vienna *Drosophila* RNAi Center and from NIG-FLY Stock Center. The following *GAL4* driver lines were used in this study: tub-GAL4 [51], ppl-GAL4 [52], r^4^-GAL4 [53], Elav-GAL4 [54] and Mef2-GAL4 [55].

### Fly food

In standard conditions flies were maintained at 25°C on medium containing agar 0.6% (w/v), semolina 3.2% (w/v), malt 6.5% (w/v), dry baker's yeast 1.8% (w/v), propionic acid 0.7% (v/v) and Nipagin (methylparaben) 2.4% (v/v). For defined nutrient studies, larvae were grown on food containing 20% (w/v) dry baker's yeast, 0.5% (w/v) agar, and 2.5% (v/v) Nipagin (methylparaben) in PBS supplemented with varying concentrations of sucrose, glucose or fructose. 1^st^ instar larvae were collected from apple juice plates (apple juice 33.33% (v/v), agar 1.75% (w/v), sugar 2.5% (w/v) and Nipagin (methylparaben) 2.0% (v/v)) and larvae were grown at controlled density (30 larvae per vial).

### Mlx antibodies

For generating anti-Mlx antibodies full-length *Drosophila mlx* cDNA was cloned into pGEX-4T2. Recombinant GST-Mlx was purified using Glutathione-agarose (Sigma). Anti-Mlx antiserum was raised by immunizing a guinea pig (Storkbio Ltd).

### Cell culture and transfections

*Drosophila* S2 cells were grown at 25°C in standard Shields and Sang M3 medium (Sigma) containing 2% of fetal bovine serum (Gibco), 1× insect medium supplement (Sigma) and penicillin/streptomycin (Gibco). The transfections were performed using Effectene (Qiagen), according to manufacturer's protocol. Expression of transfected genes was induced with 1.2 µM CuSO_4_ 24 h post-transfection.

### Pulldown and Western blotting

For detecting endogenous Mlx *in vivo*, 3^rd^ instar prewandering larvae were homogenized in Laemmli sample buffer and boiled for 5 min. Samples were resolved on SDS-PAGE and detected by Western blotting using anti-Mlx antibodies. For the pulldown experiment, cells were lysed in IP lysis buffer (10 mM Tris-HCl pH 8.0, 150 mM NaCl, 0,1% NP40) and lysates were cleared by centrifugation. Lysate protein concentration was adjusted to 1 µg/µl. 1 ml of lysate was incubated o/n with 25 µl Strep-Tactin beads (IBA). The beads were washed 5 times with IP lysis buffer, after which Laemmli sample buffer was added and samples were boiled for 5 min. Pulldown and lysate samples were resolved on SDS-PAGE, transferred to nitrocellulose and analyzed by Western blotting using anti-V5 (Invitrogen), anti-Mlx and anti-Kinesin (Cytoskeleton).

### Metabolomic analyses of small polar metabolites and lipids

Metabolomics was performed using prewandering 3^rd^ instar larvae grown on 20% yeast-5% sucrose diet. Analysis was done in four biological replicas. Data was processed using Guineu [56] and MZmine 2 [57] software packages for small polar metabolites and molecular lipids, respectively. Detailed description is available in Protocol S1.

### Metabolic assays

Metabolite assays were done using prewandering 3^rd^ instar larvae grown on 20% yeast or 20% yeast-5% sucrose diet. All analyses were done at least in four biological replicas. Glucose, trehalose and glycogen measurements were conducted as described [58], [59].

### Microarray and quantitative RT–PCR

Staged 3^rd^ instar prewandering control and *mlx^1^* mutant larvae were grown on a 20% yeast-5% sucrose diet. RNA was extracted from 3–4 larval fat bodies per sample in four biological replicas using the Nucleospin RNA XS kit (Macherey-Nagel). The Amino Allyl MessageAmp II aRNA Amplification kit (Ambion) was used for aRNA synthesis. Hybridization to Agilent *Drosophila* Gene Expression Microarray, 4×44K was performed according to the manufacturer's instructions. Data normalization and analysis was performed using R, taking advantage of packages from the Bioconductor repository. A full documentation of the data analysis is available in Protocol S1. For quantitative RT-PCR, the RevertAid H Minus First Strand cDNA Synthesis Kit (Fermentas) with random hexamer primers was used for first strand cDNA synthesis. PCR was performed using Maxima SYBR Green qPCR Master Mix (2X) (Fermentas) and analyzed on StepOnePlus (Applied Biosystems) real-time PCR system. Primer sequences are available as Table S3.

All expression profiling data is available under accession E-MTAB-699 at the ArrayExpress repository.

### Statistics

Statistical significance for each experiment (excluding metabolomics and microarray) was determined with unpaired Student's t-test with unequal variance. All quantitative data are presented as mean ± SEM for a minimum of three independent biological replicates.

## Supporting Information

Figure S1 Phenotypes of *mlx^1^* and CG3368 RNAi. (A) Relative mlx mRNA revels in control and *mlx^1^* mutant larvae measured by quantitative RT-PCR. (B) *mlx^1^* mutants die in the late pupal stage as pharate adults (C) *mlx^1^* mutants display developmental delay on regular fly food. (D) Relative CG3368 mRNA levels of control (tub-GAL4>) and CG3368 RNAi (tub-GAL4>CG3368 RNAi) larvae measured by quantitative RT-PCR. (E) Pupation kinetics of control (tub-GAL4>) CG3368 RNAi (tub-GAL4>CG3368 RNAi) larvae on 20% yeast diet with or without 15% sucrose. (F) Survival into adulthood of control (tub-GAL4>) and CG3368 RNAi (tub-GAL4>CG3368) RNAi animals grown on 20% yeast diet with or without 15% sucrose. Error bars represent ± SEM.
(TIF)

Figure S2 Comparison of the electrophoretic migration of Mlx in S2 and larval samples. Western blotting with anti-Mlx antibody. The two upper-most bands in S2 cell samples correspond to the two upper-most bands in larval lysates.
(TIF)

Figure S3 Mlx, but not CG3368, affects circulating glucose, trehalose and glycogen. (A) Ubiquitous transgenic expression of *mlx* rescues the elevated circulating glucose levels in *mlx^1^* mutants. (B) Hemolymph glucose levels in control (tub-GAL4>), CG3368 RNAi (tub-GAL4>CG3368 RNAi) and Mlx RNAi (tub-GAL4>Mlx RNAi) larvae grown on a 20% yeast-5% sucrose diet. (C) Hemolymph trehalose levels in control (tub-GAL4>), CG3368 RNAi (tub-GAL4>CG3368 RNAi) and Mlx RNAi (tub-GAL4>Mlx RNAi) larvae grown on 20% yeast-5% sucrose diet. (D) Glycogen levels in control (tub-GAL4>) and Mlx RNAi (tub-GAL4>Mlx RNAi) larvae on 20% yeast-5% sucrose diet. Error bars represent ± SEM (* p<0.05; ** p<0.01; *** p<0.001).
(TIF)

Figure S4 Effects of Mlx overexpression to pupation. Total pupation of tub-GAL4>UAS-*mlx*, Elav-GAL4>UAS-*mlx* and Mef2-GAL4>UAS-*mlx* on 20% yeast diet with or without 15% sucrose. Error bars represent ± SEM.
(TIF)

Figure S5 Aldh-III promotes sugar tolerance, but is not regulated by Cabut. (A) tub-GAL4>Aldh-III RNAi animal development to pharate pupal stage after feeding on 20% yeast diet with or without 15% sucrose. (B) Expression of transgenic *mlx* in the fat body restores survival of *mlx^1^* on sucrose-only diet. Survival of control; *mlx^1^, r^4^-GAL4*; and *mlx^1^*, r^4^-GAL4>UAS-Aldh-III larvae after 5 days on a 20% sucrose-only diet. (C) Relative *Aldh-III*, *mlx* and *mondo* mRNA levels in control (tub^ts^-GAL4>) and Cabut RNAi (tub^ts^-GAL4>Cabut RNAi) larvae. Error bars represent ± SEM (* p<0.05; *** p<0.001).
(TIF)

Protocol S1 Metabolomics and microarray analysis.
(DOC)

Table S1 Whole dataset of the microarray gene expression profiling from control and *mlx^1^* mutant fat bodies.
(PDF)

Table S2 Systematic *in vivo* RNAi screen of 103 Mlx candidate target genes for sugar sensitivity. Embryonic lethal: embryonic lethality on both diets, larval lethal: larval lethality on both diets, pupal lethal/no phenotype: equal pupation on both diets leading to pupal lethality, no phenotype: equal adult emergence on both diets, sugar sensitive: reduced pupation or adult emergence on high sugar (20% yeast-15% sucrose) diet, N/D: no data. GD and KK-lines from Vienna Drosophila RNAi Center. NIG-line from NIG-Fly Stock Center. *) Early larval lethality with yeast+15% sucrose diet, late larval lethality with yeast diet.
(PDF)

Table S3 Primer sequences used in quantitative RT-PCR.
(PDF)

## References

[1] Tsahar E, del Rio CM, Arad Z, Joy JP, Izhaki I (2005) Are the low protein requirements of nectarivorous birds the consequence of their sugary and watery diet? A test with an omnivore. Physiol Biochem Zool 78: 239–245. 15778943 10.1086/427056

[2] Welch KC Jr, Suarez RK (2007) Oxidation rate and turnover of ingested sugar in hovering Anna's (Calypte anna) and rufous (Selasphorus rufus) hummingbirds. J Exp Biol 210: 2154–2162. 17562889 10.1242/jeb.005363

[3] Lustig RH, Schmidt LA, Brindis CD (2012) Public health: The toxic truth about sugar. Nature 482: 27–29. 22297952 10.1038/482027a

[4] Tappy L, Le KA, Tran C, Paquot N (2010) Fructose and metabolic diseases: new findings, new questions. Nutrition 26: 1044–1049. 20471804 10.1016/j.nut.2010.02.014

[5] Stanhope KL (2012) Role of fructose-containing sugars in the epidemics of obesity and metabolic syndrome. Annu Rev Med 63: 329–343. 22034869 10.1146/annurev-med-042010-113026

[6] Matzkin LM, Johnson S, Paight C, Bozinovic G, Markow TA (2011) Dietary protein and sugar differentially affect development and metabolic pools in ecologically diverse Drosophila. J Nutr 141: 1127–1133. 21525254 10.3945/jn.111.138438

[7] Musselman LP, Fink JL, Narzinski K, Ramachandran PV, Hathiramani SS, et al (2011) A high-sugar diet produces obesity and insulin resistance in wild-type Drosophila. Dis Model Mech 4: 842–849. 21719444 10.1242/dmm.007948 PMC3209653

[8] Pasco MY, Leopold P (2012) High sugar-induced insulin resistance in Drosophila relies on the lipocalin Neural Lazarillo. PLoS ONE 7: e36583 doi:10.1371/journal.pone.0036583. 22567167 10.1371/journal.pone.0036583 PMC3342234

[9] Mair W, Piper MD, Partridge L (2005) Calories do not explain extension of life span by dietary restriction in Drosophila. PLoS Biol 3: e223 doi:10.1371/journal.pbio.0030223. 16000018 10.1371/journal.pbio.0030223 PMC1140680

[10] Zinke I, Schutz CS, Katzenberger JD, Bauer M, Pankratz MJ (2002) Nutrient control of gene expression in Drosophila: microarray analysis of starvation and sugar-dependent response. EMBO J 21: 6162–6173. 12426388 10.1093/emboj/cdf600 PMC137192

[11] Giaccari A, Sorice G, Muscogiuri G (2009) Glucose toxicity: the leading actor in the pathogenesis and clinical history of type 2 diabetes - mechanisms and potentials for treatment. Nutr Metab Cardiovasc Dis 19: 365–377. 19428228 10.1016/j.numecd.2009.03.018

[12] Singh DK, Winocour P, Farrington K (2011) Oxidative stress in early diabetic nephropathy: fueling the fire. Nat Rev Endocrinol 7: 176–184. 21151200 10.1038/nrendo.2010.212

[13] Saltiel AR, Kahn CR (2001) Insulin signalling and the regulation of glucose and lipid metabolism. Nature 414: 799–806. 11742412 10.1038/414799a

[14] Havula E, Hietakangas V (2012) Glucose sensing by ChREBP/MondoA-Mlx transcription factors. Semin Cell Dev Biol 10.1016/j.semcdb.2012.02.007 22406740

[15] Ma L, Robinson LN, Towle HC (2006) ChREBP*Mlx is the principal mediator of glucose-induced gene expression in the liver. J Biol Chem 281: 28721–28730. 16885160 10.1074/jbc.M601576200

[16] Stoltzman CA, Peterson CW, Breen KT, Muoio DM, Billin AN, et al (2008) Glucose sensing by MondoA:Mlx complexes: a role for hexokinases and direct regulation of thioredoxin-interacting protein expression. Proc Natl Acad Sci U S A 105: 6912–6917. 18458340 10.1073/pnas.0712199105 PMC2383952

[17] Li MV, Chen W, Harmancey RN, Nuotio-Antar AM, Imamura M, et al (2010) Glucose-6-phosphate mediates activation of the carbohydrate responsive binding protein (ChREBP). Biochem Biophys Res Commun 395: 395–400. 20382127 10.1016/j.bbrc.2010.04.028 PMC2874883

[18] Dentin R, Tomas-Cobos L, Foufelle F, Leopold J, Girard J, et al (2012) Glucose 6-phosphate, rather than xylulose 5-phosphate, is required for the activation of ChREBP in response to glucose in the liver. J Hepatol 56: 199–209. 21835137 10.1016/j.jhep.2011.07.019

[19] Stoltzman CA, Kaadige MR, Peterson CW, Ayer DE (2011) MondoA senses non-glucose sugars: regulation of thioredoxin-interacting protein (TXNIP) and the hexose transport curb. J Biol Chem 286: 38027–38034. 21908621 10.1074/jbc.M111.275503 PMC3207397

[20] Sans CL, Satterwhite DJ, Stoltzman CA, Breen KT, Ayer DE (2006) MondoA-Mlx heterodimers are candidate sensors of cellular energy status: mitochondrial localization and direct regulation of glycolysis. Mol Cell Biol 26: 4863–4871. 16782875 10.1128/MCB.00657-05 PMC1489152

[21] Jeong YS, Kim D, Lee YS, Kim HJ, Han JY, et al (2011) Integrated expression profiling and genome-wide analysis of ChREBP targets reveals the dual role for ChREBP in glucose-regulated gene expression. PLoS ONE 6: e22544 doi:10.1371/journal.pone.0022544. 21811631 10.1371/journal.pone.0022544 PMC3141076

[22] Yamashita H, Takenoshita M, Sakurai M, Bruick RK, Henzel WJ, et al (2001) A glucose-responsive transcription factor that regulates carbohydrate metabolism in the liver. Proc Natl Acad Sci U S A 98: 9116–9121. 11470916 10.1073/pnas.161284298 PMC55382

[23] Wang H, Wollheim CB (2002) ChREBP rather than USF2 regulates glucose stimulation of endogenous L-pyruvate kinase expression in insulin-secreting cells. J Biol Chem 277: 32746–32752. 12087089 10.1074/jbc.M201635200

[24] He Z, Jiang T, Wang Z, Levi M, Li J (2004) Modulation of carbohydrate response element-binding protein gene expression in 3T3-L1 adipocytes and rat adipose tissue. Am J Physiol Endocrinol Metab 287: E424–430. 15100094 10.1152/ajpendo.00568.2003

[25] Iizuka K, Bruick RK, Liang G, Horton JD, Uyeda K (2004) Deficiency of carbohydrate response element-binding protein (ChREBP) reduces lipogenesis as well as glycolysis. Proc Natl Acad Sci U S A 101: 7281–7286. 15118080 10.1073/pnas.0401516101 PMC409910

[26] Dentin R, Benhamed F, Hainault I, Fauveau V, Foufelle F, et al (2006) Liver-specific inhibition of ChREBP improves hepatic steatosis and insulin resistance in ob/ob mice. Diabetes 55: 2159–2170. 16873678 10.2337/db06-0200

[27] Billin AN, Eilers AL, Coulter KL, Logan JS, Ayer DE (2000) MondoA, a novel basic helix-loop-helix-leucine zipper transcriptional activator that constitutes a positive branch of a max-like network. Mol Cell Biol 20: 8845–8854. 11073985 10.1128/mcb.20.23.8845-8854.2000 PMC86535

[28] Herman MA, Peroni OD, Villoria J, Schon MR, Abumrad NA, et al (2012) A novel ChREBP isoform in adipose tissue regulates systemic glucose metabolism. Nature 484: 333–338. 22466288 10.1038/nature10986 PMC3341994

[29] Peyrefitte S, Kahn D, Haenlin M (2001) New members of the Drosophila Myc transcription factor subfamily revealed by a genome-wide examination for basic helix-loop-helix genes. Mech Dev 104: 99–104. 11404084 10.1016/s0925-4773(01)00360-4

[30] McFerrin LG, Atchley WR (2011) Evolution of the Max and Mlx networks in animals. Genome Biol Evol 3: 915–937. 21859806 10.1093/gbe/evr082 PMC3177325

[31] Becker A, Schloder P, Steele JE, Wegener G (1996) The regulation of trehalose metabolism in insects. Experientia 52: 433–439. 8706810 10.1007/BF01919312

[32] Ishii S, Iizuka K, Miller BC, Uyeda K (2004) Carbohydrate response element binding protein directly promotes lipogenic enzyme gene transcription. Proc Natl Acad Sci U S A 101: 15597–15602. 15496471 10.1073/pnas.0405238101 PMC524841

[33] Sassu ED, McDermott JE, Keys BJ, Esmaeili M, Keene AC, et al (2012) Mio/dChREBP coordinately increases fat mass by regulating lipid synthesis and feeding behavior in Drosophila. Biochem Biophys Res Commun 10.1016/j.bbrc.2012.08.028 PMC3445662 22910416

[34] Singh S, Brocker C, Koppaka V, Chen Y, Jackson BC, et al (2012) Aldehyde dehydrogenases in cellular responses to oxidative/electrophilic stress. Free Radic Biol Med 56C: 89–101. 23195683 10.1016/j.freeradbiomed.2012.11.010 PMC3631350

[35] Townsend AJ, Leone-Kabler S, Haynes RL, Wu Y, Szweda L, et al (2001) Selective protection by stably transfected human ALDH3A1 (but not human ALDH1A1) against toxicity of aliphatic aldehydes in V79 cells. Chem Biol Interact 130–132: 261–273. 10.1016/s0009-2797(00)00270-2 11306050

[36] Pappa A, Chen C, Koutalos Y, Townsend AJ, Vasiliou V (2003) Aldh3a1 protects human corneal epithelial cells from ultraviolet- and 4-hydroxy-2-nonenal-induced oxidative damage. Free Radic Biol Med 34: 1178–1189. 12706498 10.1016/s0891-5849(03)00070-4

[37] Lassen N, Pappa A, Black WJ, Jester JV, Day BJ, et al (2006) Antioxidant function of corneal ALDH3A1 in cultured stromal fibroblasts. Free Radic Biol Med 41: 1459–1469. 17023273 10.1016/j.freeradbiomed.2006.08.009

[38] Yu HS, Oyama T, Isse T, Kitakawa K, Ogawa M, et al (2009) Characteristics of aldehyde dehydrogenase 2 (Aldh2) knockout mice. Toxicol Mech Methods 19: 535–540. 19874182 10.3109/15376510903401708

[39] Crabb DW, Edenberg HJ, Bosron WF, Li TK (1989) Genotypes for aldehyde dehydrogenase deficiency and alcohol sensitivity. The inactive ALDH2(2) allele is dominant. J Clin Invest 83: 314–316. 2562960 10.1172/JCI113875 PMC303676

[40] Yalcin A, Telang S, Clem B, Chesney J (2009) Regulation of glucose metabolism by 6-phosphofructo-2-kinase/fructose-2,6-bisphosphatases in cancer. Exp Mol Pathol 86: 174–179. 19454274 10.1016/j.yexmp.2009.01.003

[41] Deprez J, Vertommen D, Alessi DR, Hue L, Rider MH (1997) Phosphorylation and activation of heart 6-phosphofructo-2-kinase by protein kinase B and other protein kinases of the insulin signaling cascades. J Biol Chem 272: 17269–17275. 9211863 10.1074/jbc.272.28.17269

[42] Mouton V, Toussaint L, Vertommen D, Gueuning MA, Maisin L, et al (2010) Heart 6-phosphofructo-2-kinase activation by insulin requires PKB (protein kinase B), but not SGK3 (serum- and glucocorticoid-induced protein kinase 3). Biochem J 431: 267–275. 20687898 10.1042/BJ20101089

[43] Guertin DA, Guntur KV, Bell GW, Thoreen CC, Sabatini DM (2006) Functional genomics identifies TOR-regulated genes that control growth and division. Curr Biol 16: 958–970. 16713952 10.1016/j.cub.2006.03.084

[44] Bulow MH, Aebersold R, Pankratz MJ, Junger MA (2010) The Drosophila FoxA ortholog Fork head regulates growth and gene expression downstream of Target of rapamycin. PLoS ONE 5: e15171 doi:10.1371/journal.pone.0015171. 21217822 10.1371/journal.pone.0015171 PMC3013099

[45] Munoz-Descalzo S, Terol J, Paricio N (2005) Cabut, a C2H2 zinc finger transcription factor, is required during Drosophila dorsal closure downstream of JNK signaling. Dev Biol 287: 168–179. 16198331 10.1016/j.ydbio.2005.08.048

[46] Rodriguez I (2011) Drosophila TIEG is a modulator of different signalling pathways involved in wing patterning and cell proliferation. PLoS ONE 6: e18418 doi:10.1371/journal.pone.0018418. 21494610 10.1371/journal.pone.0018418 PMC3072976

[47] Neve B, Fernandez-Zapico ME, Ashkenazi-Katalan V, Dina C, Hamid YH, et al (2005) Role of transcription factor KLF11 and its diabetes-associated gene variants in pancreatic beta cell function. Proc Natl Acad Sci U S A 102: 4807–4812. 15774581 10.1073/pnas.0409177102 PMC554843

[48] Iizuka K, Takeda J, Horikawa Y (2011) Kruppel-like factor-10 is directly regulated by carbohydrate response element-binding protein in rat primary hepatocytes. Biochem Biophys Res Commun 412: 638–643. 21856285 10.1016/j.bbrc.2011.08.016

[49] Guillaumond F, Grechez-Cassiau A, Subramaniam M, Brangolo S, Peteri-Brunback B, et al (2010) Kruppel-like factor KLF10 is a link between the circadian clock and metabolism in liver. Mol Cell Biol 30: 3059–3070. 20385766 10.1128/MCB.01141-09 PMC2876690

[50] Bouteldja N, Timson DJ (2010) The biochemical basis of hereditary fructose intolerance. J Inherit Metab Dis 33: 105–112. 20162364 10.1007/s10545-010-9053-2

[51] Lee T, Luo L (1999) Mosaic analysis with a repressible cell marker for studies of gene function in neuronal morphogenesis. Neuron 22: 451–461. 10197526 10.1016/s0896-6273(00)80701-1

[52] Zinke I, Kirchner C, Chao LC, Tetzlaff MT, Pankratz MJ (1999) Suppression of food intake and growth by amino acids in Drosophila: the role of pumpless, a fat body expressed gene with homology to vertebrate glycine cleavage system. Development 126: 5275–5284. 10556053 10.1242/dev.126.23.5275

[53] Lee G, Park JH (2004) Hemolymph sugar homeostasis and starvation-induced hyperactivity affected by genetic manipulations of the adipokinetic hormone-encoding gene in Drosophila melanogaster. Genetics 167: 311–323. 15166157 10.1534/genetics.167.1.311 PMC1470856

[54] Luo L, Liao YJ, Jan LY, Jan YN (1994) Distinct morphogenetic functions of similar small GTPases: Drosophila Drac1 is involved in axonal outgrowth and myoblast fusion. Genes Dev 8: 1787–1802. 7958857 10.1101/gad.8.15.1787

[55] Ranganayakulu G, Elliott DA, Harvey RP, Olson EN (1998) Divergent roles for NK-2 class homeobox genes in cardiogenesis in flies and mice. Development 125: 3037–3048. 9671578 10.1242/dev.125.16.3037

[56] Castillo S, Mattila I, Miettinen J, Oresic M, Hyotylainen T (2011) Data analysis tool for comprehensive two-dimensional gas chromatography/time-of-flight mass spectrometry. Anal Chem 83: 3058–3067. 21434611 10.1021/ac103308x

[57] Pluskal T, Castillo S, Villar-Briones A, Oresic M (2010) MZmine 2: modular framework for processing, visualizing, and analyzing mass spectrometry-based molecular profile data. BMC Bioinformatics 11: 395. 20650010 10.1186/1471-2105-11-395 PMC2918584

[58] Zhang W, Thompson BJ, Hietakangas V, Cohen SM (2011) MAPK/ERK signaling regulates insulin sensitivity to control glucose metabolism in Drosophila. PLoS Genet 7: e1002429 doi:10.1371/journal.pgen.1002429. 22242005 10.1371/journal.pgen.1002429 PMC3248469

[59] Parrou JL, Francois J (1997) A simplified procedure for a rapid and reliable assay of both glycogen and trehalose in whole yeast cells. Anal Biochem 248: 186–188. 9177741 10.1006/abio.1997.2138

