# Supplementary figures and images for: Mondo/ChREBP-Mlx-Regulated Transcriptional Network Is Essential for Dietary Sugar Tolerance in Drosophila
Source: PLoS Genet. 2013 Apr 4;9(4):e1003438. doi: 10.1371/journal.pgen.1003438 (PMC3616910; doi:10.1371/journal.pgen.1003438)

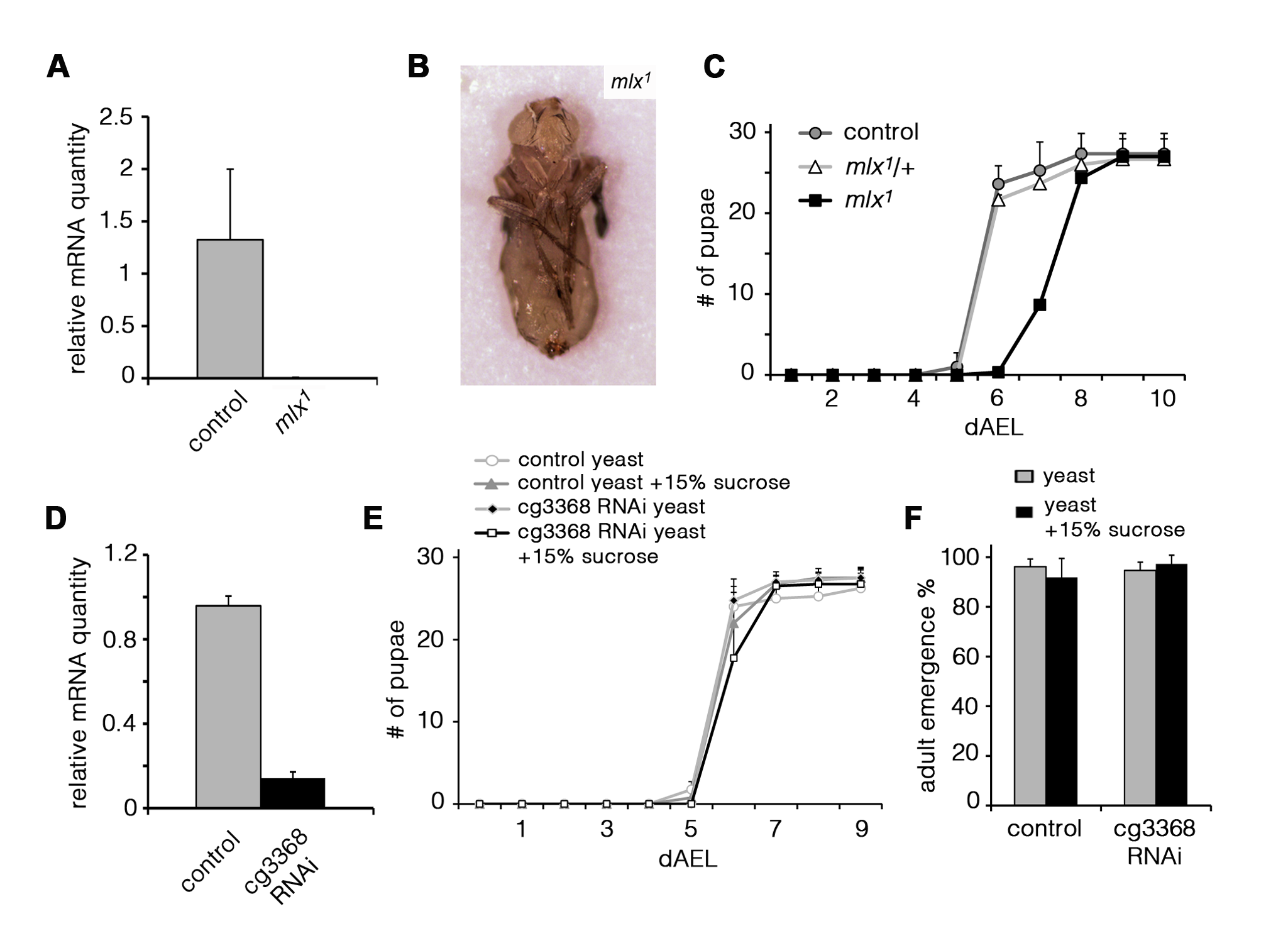

Supplement: Figure S1 — Phenotypes of mlx1 and CG3368 RNAi. (A) Relative mlx mRNA revels in control and mlx1 mutant larvae measured by quantitative RT-PCR. (B) mlx1 mutants die in the late pupal stage as pharate adults (C) mlx1 mutants display developmental delay on regular fly food. (D) Relative CG3368 mRNA levels of control (tub-GAL4>) and CG3368 RNAi (tub-GAL4>CG3368 RNAi) larvae measured by quantitative RT-PCR. (E) Pupation kinetics of control (tub-GAL4>) CG3368 RNAi (tub-GAL4>CG3368 RNAi) larvae on 20% yeast diet with or without 15% sucrose. (F) Survival into adulthood of control (tub-GAL4>) and CG3368 RNAi (tub-GAL4>CG3368) RNAi animals grown on 20% yeast diet with or without 15% sucrose. Error bars represent ± SEM. (TIF) [file pgen.1003438.s001.tif]

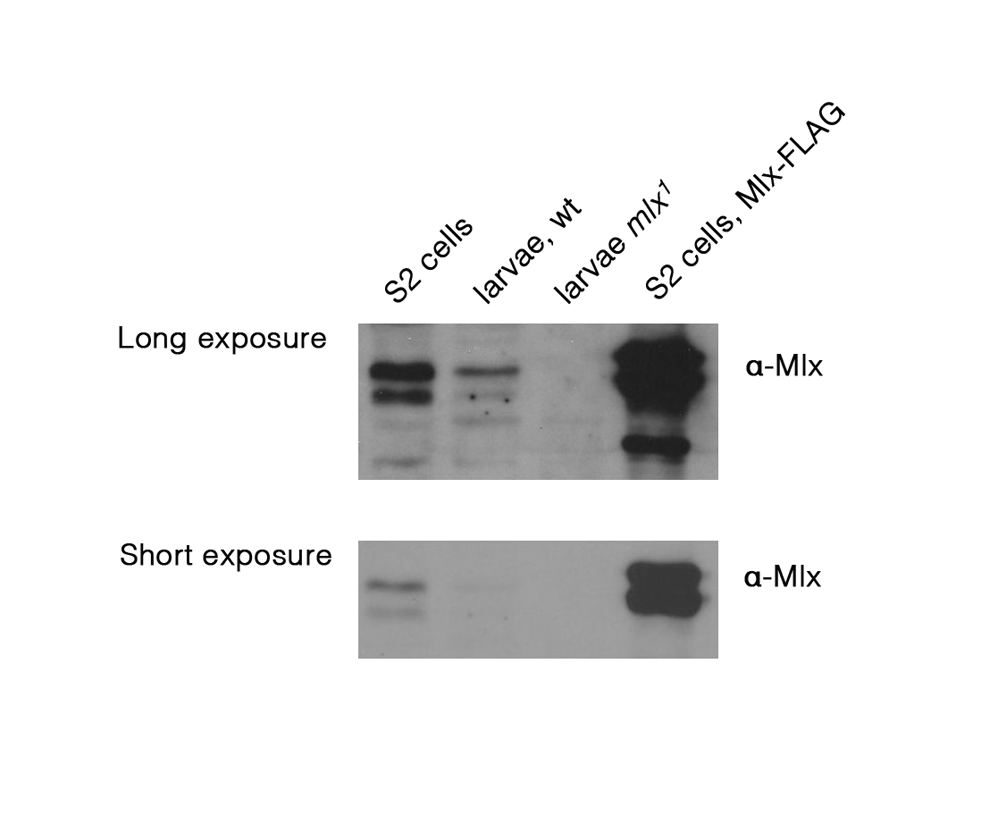

Supplement: Figure S2 — Comparison of the electrophoretic migration of Mlx in S2 and larval samples. Western blotting with anti-Mlx antibody. The two upper-most bands in S2 cell samples correspond to the two upper-most bands in larval lysates. (TIF) [file pgen.1003438.s002.tif]

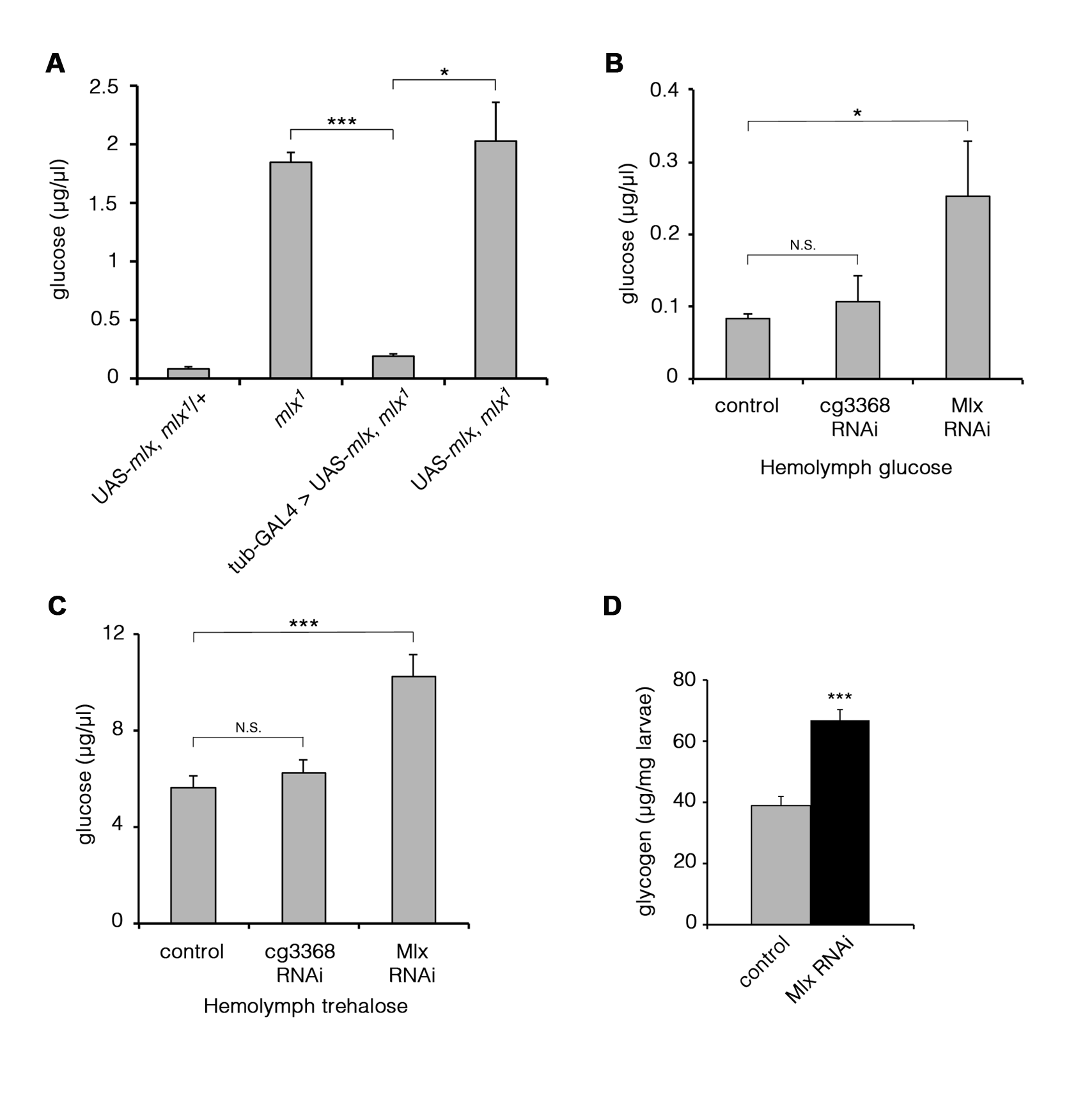

Supplement: Figure S3 — Mlx, but not CG3368, affects circulating glucose, trehalose and glycogen. (A) Ubiquitous transgenic expression of mlx rescues the elevated circulating glucose levels in mlx1 mutants. (B) Hemolymph glucose levels in control (tub-GAL4>), CG3368 RNAi (tub-GAL4>CG3368 RNAi) and Mlx RNAi (tub-GAL4>Mlx RNAi) larvae grown on a 20% yeast-5% sucrose diet. (C) Hemolymph trehalose levels in control (tub-GAL4>), CG3368 RNAi (tub-GAL4>CG3368 RNAi) and Mlx RNAi (tub-GAL4>Mlx RNAi) larvae grown on 20% yeast-5% sucrose diet. (D) Glycogen levels in control (tub-GAL4>) and Mlx RNAi (tub-GAL4>Mlx RNAi) larvae on 20% yeast-5% sucrose diet. Error bars represent ± SEM (* p<0.05; ** p<0.01; *** p<0.001). (TIF) [file pgen.1003438.s003.tif]

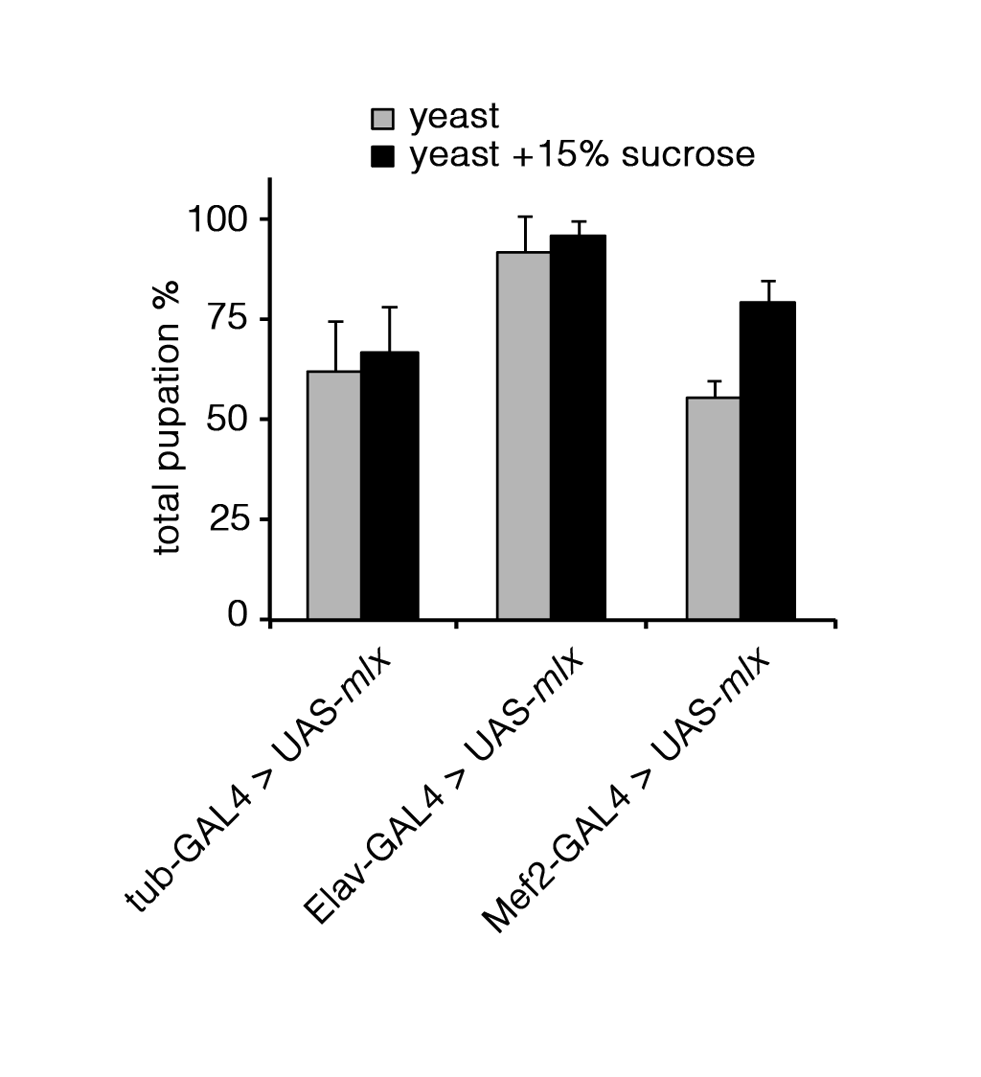

Supplement: Figure S4 — Effects of Mlx overexpression to pupation. Total pupation of tub-GAL4>UAS-mlx, Elav-GAL4>UAS-mlx and Mef2-GAL4>UAS-mlx on 20% yeast diet with or without 15% sucrose. Error bars represent ± SEM. (TIF) [file pgen.1003438.s004.tif]

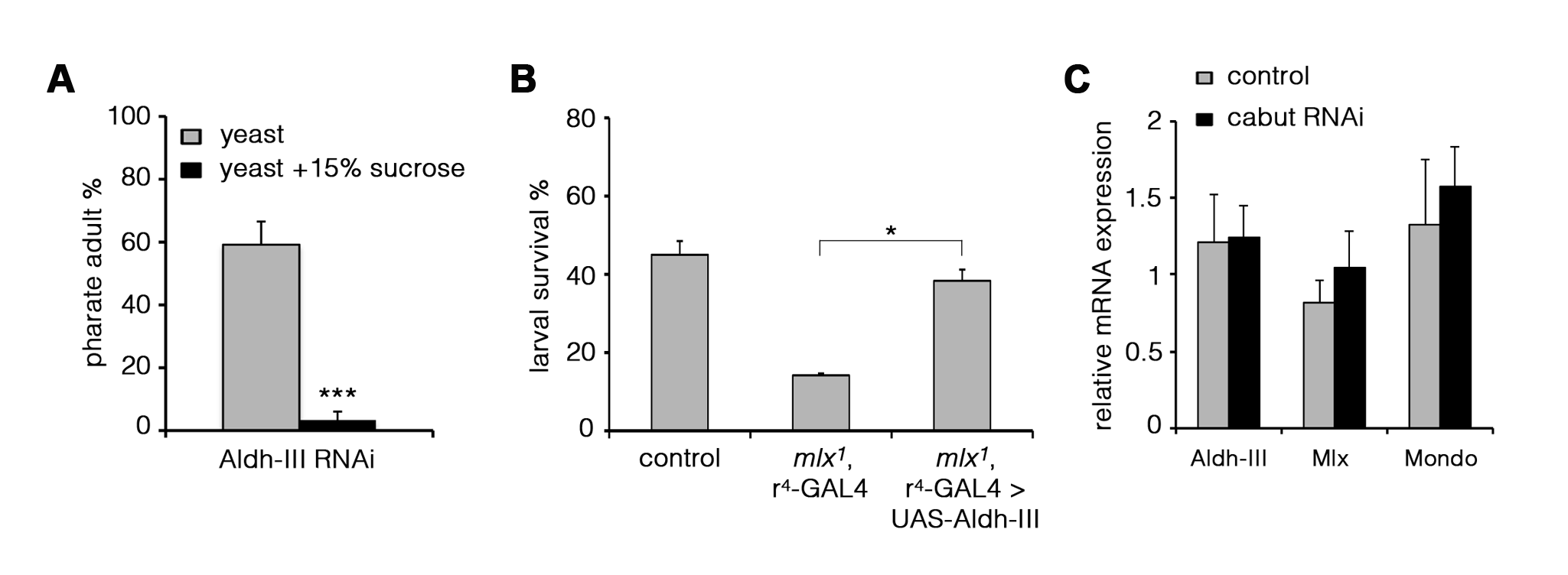

Supplement: Figure S5 — Aldh-III promotes sugar tolerance, but is not regulated by Cabut. (A) tub-GAL4>Aldh-III RNAi animal development to pharate pupal stage after feeding on 20% yeast diet with or without 15% sucrose. (B) Expression of transgenic mlx in the fat body restores survival of mlx1 on sucrose-only diet. Survival of control; mlx1, r4-GAL4; and mlx1, r4-GAL4>UAS-Aldh-III larvae after 5 days on a 20% sucrose-only diet. (C) Relative Aldh-III, mlx and mondo mRNA levels in control (tubts-GAL4>) and Cabut RNAi (tubts-GAL4>Cabut RNAi) larvae. Error bars represent ± SEM (* p<0.05; *** p<0.001). (TIF) [file pgen.1003438.s005.tif]
