# Supplementary material for: Mondo/ChREBP-Mlx-Regulated Transcriptional Network Is Essential for Dietary Sugar Tolerance in Drosophila
Source: PLoS Genet. 2013 Apr 4;9(4):e1003438. doi: 10.1371/journal.pgen.1003438 (PMC3616910; doi:10.1371/journal.pgen.1003438)
Supplement: Protocol S1 — Metabolomics and microarray analysis. (DOC) [file pgen.1003438.s006.doc]

**SUPPLEMENTAL EXPERIMENTAL PROCEDURES**

**Metabolomics**

Sample preparation, step 1, homogenisation:

200 µl 0.9 % NaCl solution was added to the larvae samples (ca. 30 mg), 3 grinding balls were added and they were homogenised 2 min at 20Hz with Retsch grinding mill.

GCxGC-TOFMS method for small polar metabolites

## *Sample preparation*

400 µl methanol and 10 µl internal standard mixture (C17:0 (93.3 mg/l), valine-d (18.5 mg/l) and succinic acid-d4 (31.5 mg/l)) were added to 50 µl of sample homogenate. The samples were vortex mixed (2 min at 20 Hz). After 30 minutes at room temperature the samples were centrifuged for 5 min at 10000 rpm. The supernatant was moved to a GC vial and evaporated to dryness under nitrogen. The samples were trimethylsilylated with 25 µl MOX (45°C, 60 min) and 25 µl MSTFA (45°C, 60 min) and 5 µl of retention index solution in hexane was added to samples (150 mg/l C11, C15, C17, C21 and C25 alkanes).

## *Instrument*

For the analysis, a Leco Pegasus 4D GC×GC-TOFMS instrument (Leco Corp., St. Joseph, MI) equipped with a cryogenic modulator was used. The GC part of the instrument was an Agilent 6890 gas chromatograph (Agilent Technologies, Palo Alto, CA), equipped with split/splitless injector. The first-dimension chromatographic column was a 10-m RTX-5 capillary column with an internal diameter of 0.18 mm and a stationary-phase film thickness of 0.20 μm, and the second-dimension chromatographic column was a 1.5 m BPX-50 capillary column with an internal diameter of 100 μm and a film thickness of 0.1 μm. A methyl deactivated retention gap (3 m x 0.53 mm i.d.) was used in the front of the first column. High-purity helium was used as the carrier gas at a constant pressure mode (39.6 psig). A 5-s separation time was used in the second dimension. The MS spectra were measured at 45 – 700 amu with 100 spectra/sec. For the injection, a pulsed splitless injection (0.5 µl) at 240 °C was utilized, with pulse pressure of 55 psig for 1 minute. The temperature program was as follows: the first-dimension column oven ramp began at 40 °C with a 2 min hold after which the temperature was programmed to 295 °C at a rate of 7 °C/min and then held at this temperature for 3 min. The second-dimension column temperature was maintained 20 °C higher than the corresponding first-dimension column. The programming rate and hold times were the same for the two columns.

*Data processing*

ChromaTOF vendor software (LECO) was used for within-sample data processing, and in house made software Guineu was used for alignment, normalisation and peak matching across samples. The peaks were first filtered based on number of detected peaks in the total profile of all sample runs. The normalization was performed by correction for internal standards and protein amount. 27 of the metabolites (Table I) were checked manually in each sample for correct integration and identification. Other mass spectra from the GCxGC-TOF/MS analysis were searched against The Palisade Complete Mass Spectral Library, 600K Edition (Palisade Mass Spectrometry, Ithaca, NY), which includes all spectra available from the NIST 2002 and Wiley registry collections as well as 150,000 other spectra. The matches to reference spectra are based on a weighted dot product of the two spectra, with higher m/z peaks having more weight than the lower. A similarity value is assigned between 0 and 999, with 999 being a perfect match.

**Table I.** Quantified metabolites, and the range of calibration with GCxGC-TOFMS.

| **Compound** | Min | Max |
| --- | --- | --- |
| Pyruvic acid, 2TMS | 15.7 | 524.4 |
| Alanine, 2TMS | 27.0 | 900.8 |
| 3-Hydroxybutyric acid, 2TMS | 41.9 | 1397.6 |
| Valine, 2TMS | 35.6 | 1185.0 |
| Leucine, 2TMS | 39.8 | 1326.6 |
| Isoleucine, 2TMS | 39.8 | 1326.6 |
| Proline, 2TMS | 34.9 | 1163.8 |
| Glycine, 3TMS | 22.8 | 759.0 |
| Succinic acid, 2TMS | 16.3 | 543.3 |
| Fumaric acid, 2TMS | 15.7 | 522.4 |
| Serine, 3TMS | 31.9 | 1062.7 |
| Threonine, 3TMS | 36.1 | 1204.2 |
| Malic acid, 3TMS | 15.3 | 508.5 |
| Methionine, 2TMS | 45.3 | 1508.6 |
| Aspartic acid, 3TMS | 40.4 | 1345.8 |
| Alpha-ketoglutaric acid, TMS | 3.3 | 109.0 |
| Phenylalanine, 2TMS | 50.1 | 1670.4 |
| Glutamic acid, 3TMS | 44.6 | 1487.4 |
| Ornithine, 4TMS | 49.5 | 1649.9 |
| Citric acid, 4TMS | 73.8 | 2459.8 |
| Tyrosine, 3TMS | 55.0 | 1832.2 |
| Palmitic acid, TMS | 42.9 | 1429.3 |
| Linoleic acid, TMS | 47.8 | 1593.9 |
| Oleic acid, TMS | 49.7 | 1656.6 |
| Stearic acid, TMS | 41.5 | 1383.1 |
| Arachidonic acid, TMS | 47.0 | 1567.5 |
| Cholesterol, TMS | 178.5 | 5950.0 |

UPLC-MS for molecular lipids (global lipidomics)

*Sample preparation*

For the UPLC-QTOFMS analyses, a standard mixture 1 (10 μl) containing PC(17:0/0:0), PC(17:0/17:0), PE(17:0/17:0) and Cer(d18:1/17:0), (Avanti Polar Lipids, Inc.) and TG(17:0/17:0/17:0) (Larodan Fine Chemicals) was added to 5 µl of sample homogenate. HPLC-grade chloroform and methanol (2:1; 100 µl) was added to the samples, and the samples were vortexed for 2 min and allowed to stand for 30 min. Subsequently, samples were centrifuged and the lower phase (60 µl) was collected and 20 µl of internal standard mixture 2 is added. The internal standard mixture 2 contained the labelled lipids PC (16:1/0:0-D_3_), PC (16:1/16:1-D_6_) and TG(16:0/16:0/16:0-^13^C3) .

*Instrumental conditions*

The larvae extracts were analyzed on a Waters Q-Tof Premier mass spectrometer combined with an Acquity Ultra Performance LC^TM^ (UPLC). The column (at 50 °C) was an Acquity UPLC^TM^ BEH C18 2.1 × 100 mm with 1.7 μm particles. The solvent system included ultrapure water (1% 1 M NH_4_Ac, 0.1% HCOOH) and (B) LC/MS grade acetonitrile/isopropanol (1:1, 1% 1M NH_4_Ac, 0.1% HCOOH). The gradient started from 65% A / 35% B, reached 80% B in 2 min, 100% B in 7 min, and remained there for 7 min. The flow rate was 0.400 ml/min and the injected amount was 2.0 μl (Acquity Sample Organizer, at 10 °C). Reserpine was used as the lock spray reference compound. The lipid profiling was carried out using ESI in positive mode and the data was collected at a mass range of m/z 300-1200 with scan duration of 0.2 sec.

*Data processing*

The data processing using MZmine2 software included alignment of peaks, peak integration, normalization, and peak identification. Lipids were identified using an internal spectral library. The data was normalized using one or more internal standards representatives of each class of lipid present in the samples: the intensity of each identified lipid was normalized by dividing it with the intensity of its corresponding standard and multiplying it by the concentration of the standard. All monoacyl lipids except cholesterol esters, such as monoacylglycerols and monoacylglycerophospholipids, were normalized with PC(17:0/0:0), all diacyl lipids except ethanolamine phospholipids were normalized with PC(17:0/17:0), all ceramides with Cer(d18:1/17:0), all diacyl ethanolamine phospholipids with PE(17:0/17:0), and TG and cholesterol esters were normalized with TG(17:0/17:0/17:0). Other (unidentified) molecular species were normalized with PC(17:0/0:0) for retention time < 300 s, PC(17:0/17:0) for retention time between 300 s and 410 s, and TG(17:0/17:0/17:0) for higher retention times. Finally, the lipids were further normalized by dividing the normalized lipid concentration with the protein content of the sample.

*Proteins*

5 µl of sample homogenate was mixed with 95µl PBS-buffer and the mixture was further diluted with the PBS buffer. The final dilution was 1:500 (v/v). The protein determination was done with the Micro BCA™ Protein Assay Reagent Kit. The determination is based on bicinchoninic acid (BCA) for the colorimetric detection and quantitation of total protein. This method combines reduction of Cu+2 to Cu+1 by protein in an alkaline medium (the biuret reaction) with the highly sensitive and selective colorimetric detection of the cuprous cation (Cu+1) using a reagent containing bicinchoninic acid. Albumin was used for protein standard samples.

**Microarray and data analysis**

Raw foreground and background intensities for the three different channels of each microarray were imported into R using limma package . Background subtraction and intensity normalization was performed separately for each channel with the justvsn function from the Bioconductor VSN package .

To identify differentially expressed genes, we fit a linear model including the experimental factors ”genotype” and ”food” to the dataset by applying the lm- Fit and eBayes functions from the limma package. 193 genes differentially expressed between wildtype and *mlx^1^* mutant animals were identified by calculating adjusted p-values for each gene according to Benjamini and Hochberg . For genes represented by multiple cDNA probes on the microarray, only the result with the smallest adjusted p-value was retained.

To identify functional categories of genes significantly affected by loss of *mlx*, we performed gene-set enrichment analysis (GSEA) using annotation from the KEGG database by employing the Bioconductor HTSanalyzeR package . Significantly enriched KEGG categories were selected by applying an adjusted p-value cutoff of 0.05.

**SUPPLEMENTAL REFERENCES**

1. Castillo S, Mattila I, Miettinen J, Oresic M, Hyotylainen T (2011) Data analysis tool for comprehensive two-dimensional gas chromatography/time-of-flight mass spectrometry. Anal Chem 83: 3058-3067.

2. Pluskal T, Castillo S, Villar-Briones A, Oresic M (2010) MZmine 2: modular framework for processing, visualizing, and analyzing mass spectrometry-based molecular profile data. BMC Bioinformatics 11: 395.

3. Smyth GK (2004) Linear models and empirical bayes methods for assessing differential expression in microarray experiments. Stat Appl Genet Mol Biol 3: Article3.

4. Huber W, von Heydebreck A, Sultmann H, Poustka A, Vingron M (2002) Variance stabilization applied to microarray data calibration and to the quantification of differential expression. Bioinformatics 18 Suppl 1: S96-104.

5. Benjamini Y, Hochberg Y (1995) Controlling the False Discovery Rate - a Practical and Powerful Approach to Multiple Testing. Journal of the Royal Statistical Society Series B-Methodological 57: 289-300.

6. Kotera M, Hirakawa M, Tokimatsu T, Goto S, Kanehisa M (2012) The KEGG databases and tools facilitating omics analysis: latest developments involving human diseases and pharmaceuticals. Methods Mol Biol 802: 19-39.

7. Wang X, Terfve C, Rose JC, Markowetz F (2011) HTSanalyzeR: an R/Bioconductor package for integrated network analysis of high-throughput screens. Bioinformatics 27: 879-880.
