# Supplementary material for: Mondo/ChREBP-Mlx-Regulated Transcriptional Network Is Essential for Dietary Sugar Tolerance in Drosophila
Source: PLoS Genet. 2013 Apr 4;9(4):e1003438. doi: 10.1371/journal.pgen.1003438 (PMC3616910; doi:10.1371/journal.pgen.1003438)
Supplement: Table S1 — Whole dataset of the microarray gene expression profiling from control and mlx1 mutant fat bodies. (PDF) [file pgen.1003438.s007.pdf]

***Supplemental table 1.***

| ProbeID | EntrezGeneID | GeneSymbol | logFC | P.Value | adj.P.Val |
|---|---|---|---|---|---|
| A_09_P072311 | 43293 | mlx | -4.44 | 3.85e-06 | 0.00392 |
| A_09_P056476 | 33224 | cbt | -4.02 | 3.24e-06 | 0.00388 |
| A_09_P072306 | 43292 | CG3368 | -3.93 | 9.48e-07 | 0.00273 |
| A_09_P024801 | 35687 | CG1600 | -3.75 | 4.29e-05 | 0.0159 |
| A_09_P020546 | 34086 | CG7231 | -3.74 | 2.05e-07 | 0.00112 |
| A_09_P018321 | 33348 | mRpL48 | -3.7 | 3.26e-06 | 0.00388 |
| A_09_P005646 | 35946 | Cyp4p2 | -3.57 | 1e-06 | 0.00273 |
| A_09_P063236 | 50082 | CG3348 | -3.52 | 1.73e-06 | 0.00344 |
| A_09_P018531 | 33421 | CG3609 | -3.27 | 1.72e-05 | 0.00972 |
| A_09_P061061 | 43512 | CG12068 | -3.05 | 0.000105 | 0.0232 |
| A_09_P188560 | 318935 | CG31775 | -3.01 | 0.000386 | 0.0441 |
| A_09_P021726 | 34471 | CG17108 | -2.89 | 0.000137 | 0.0253 |
| A_09_P068256 | 31779 | CG15347 | -2.88 | 1.89e-05 | 0.0101 |
| A_09_P008501 | 36891 | CG6426 | -2.78 | 4.91e-05 | 0.0165 |
| A_09_P047741 | 4379870 | CG34141 | -2.71 | 4.26e-06 | 0.00404 |
| A_09_P032266 | 44381 | Tim17b2 | -2.7 | 6.1e-05 | 0.0183 |
| A_09_P003676 | 5740866 | CG34301 | -2.67 | 1.15e-05 | 0.00719 |
| A_09_P108115 | 32696 | Arpc3B | -2.65 | 0.00037 | 0.0432 |
| A_09_P103970 | 46017 | l(2)01289 | -2.55 | 4.33e-05 | 0.0159 |
| A_09_P079021 | 35139 | CG17322 | -2.47 | 2.64e-06 | 0.00371 |
| A_09_P144790 | 33824 | Gpdh | -2.46 | 1.18e-05 | 0.00724 |
| A_09_P209885 | 5740446 | Rgk3 | -2.43 | 4.71e-05 | 0.0163 |
| A_09_P192980 | 32924 | CG8034 | -2.33 | 2.23e-05 | 0.0109 |
| A_09_P068861 | 42211 | CG7675 | -2.32 | 0.000112 | 0.0232 |
| A_09_P003006 | 5740685 | CG34165 | -2.24 | 5.7e-05 | 0.0179 |
| A_09_P008941 | 37053 | CG5773 | -2.23 | 5.23e-05 | 0.0171 |
| A_09_P060741 | 43419 | CG14519 | -2.23 | 0.000175 | 0.0279 |
| A_09_P011751 | 43814 | bt | -2.22 | 6.26e-06 | 0.00515 |
| A_09_P167600 | 35790 | Lpin | -2.18 | 0.00045 | 0.0482 |
| A_09_P136070 | 326329 | CG33199 | -2.17 | 0.000144 | 0.0259 |
| A_09_P005567 | 35919 | CG13741 | -2.12 | 8.7e-05 | 0.0212 |
| A_09_P101715 | 5740644 | CG34288 | -2.1 | 2.22e-05 | 0.0109 |
| A_09_P011846 | 45396 | E5 | -2.09 | 0.000136 | 0.0253 |
| A_09_P150455 | 43616 | aralar1 | -2.04 | 4.98e-05 | 0.0166 |
| A_09_P020151 | 33955 | CG11236 | -2.04 | 4.28e-05 | 0.0159 |
| A_09_P025361 | 35898 | CG8229 | -2.04 | 0.000106 | 0.0232 |
| A_09_P022806 | 35003 | CG13277 | -2.01 | 1.1e-07 | 0.00112 |
| A_09_P078731 | 42849 | CG5991 | -1.97 | 0.000135 | 0.0253 |
| A_09_P073236 | 41198 | CG12946 | -1.97 | 0.000412 | 0.0461 |
| A_09_P064541 | 317813 | CG18789 | -1.96 | 4.57e-05 | 0.0161 |
| A_09_P221345 | 37116 | Dp1 | -1.96 | 4e-06 | 0.00396 |
| A_09_P124315 | 36532 | CG6357 | -1.94 | 6.83e-05 | 0.0193 |
| A_09_P043201 | 35184 | Lim3 | -1.93 | 3.76e-06 | 0.00392 |
| A_09_P220650 | 39084 | CG3982 | -1.92 | 0.000126 | 0.0246 |
| A_09_P168970 | 117369 | desat1 | -1.91 | 4.7e-05 | 0.0163 |
| A_09_P058541 | 246627 | CG30463 | -1.88 | 6.53e-05 | 0.0188 |
| A_09_P128570 | 50300 | CG13315 | -1.87 | 7.21e-05 | 0.0198 |
| A_09_P007966 | 36700 | CG8157 | -1.86 | 0.000195 | 0.0291 |
| A_09_P012626 | 45398 | Aldh-III | -1.83 | 5.3e-06 | 0.00472 |
| A_09_P012871 | 46234 | fbl | -1.82 | 0.000151 | 0.0267 |
| A_09_P189195 | 43892 | sif | -1.82 | 3.64e-05 | 0.0151 |
| A_09_P063536 | 50239 | CG13235 | -1.79 | 0.000327 | 0.0403 |
| A_09_P021656 | 34447 | CG7384 | -1.78 | 7.87e-05 | 0.0205 |
| A_09_P197250 | 40273 | CG32425 | -1.74 | 0.00013 | 0.0253 |

| | | | | | |
|---|---|---|---|---|---|
| A_09_P042626 | 33172 | Gs1 | -1.73 | 0.000254 | 0.034 |
| A_09_P010076 | 31939 | Yp1 | -1.71 | 0.000365 | 0.0429 |
| A_09_P042821 | 42852 | Hsp68 | -1.7 | 8e-05 | 0.0205 |
| A_09_P178020 | 5740638 | CR41610 | -1.68 | 0.000164 | 0.0273 |
| A_09_P076656 | 3771960 | waw | -1.66 | 0.000276 | 0.0361 |
| A_09_P040561 | 32962 | CG14222 | -1.65 | 0.000343 | 0.0416 |
| A_09_P046696 | 5740691 | CG41265 | -1.62 | 0.000402 | 0.0456 |
| A_09_P125465 | 39675 | Eip71CD | -1.62 | 4.46e-05 | 0.0159 |
| A_09_P071756 | 43104 | CG4774 | -1.6 | 3.84e-05 | 0.0152 |
| A_09_P069671 | 42462 | CG17270 | -1.6 | 0.000189 | 0.0287 |
| A_09_P025456 | 37248 | CG15120 | -1.58 | 0.000294 | 0.0377 |
| A_09_P006611 | 36276 | CG13188 | -1.58 | 0.000188 | 0.0287 |
| A_09_P064231 | 117347 | Gr39b | -1.56 | 4.07e-05 | 0.0158 |
| A_09_P027746 | 37980 | Ance-5 | -1.55 | 0.00021 | 0.0304 |
| A_09_P014846 | 317902 | CG32189 | -1.54 | 4.01e-05 | 0.0158 |
| A_09_P141770 | 318102 | beltless | -1.54 | 0.000195 | 0.0291 |
| A_09_P060021 | 40567 | CG31522 | -1.53 | 0.000102 | 0.0231 |
| A_09_P070621 | 42762 | CG4408 | -1.53 | 0.000399 | 0.0454 |
| A_09_P018751 | 33483 | CG18557 | -1.44 | 0.000242 | 0.0332 |
| A_09_P030021 | 36753 | Strn-Mlck | -1.43 | 5.11e-05 | 0.0169 |
| A_09_P028356 | 38186 | CG13917 | -1.42 | 2.11e-05 | 0.0108 |
| A_09_P023176 | 35115 | Cyp310a1 | -1.42 | 0.000138 | 0.0253 |
| A_09_P020016 | 33907 | CG9542 | -1.41 | 0.000105 | 0.0232 |
| A_09_P077281 | 31258 | CG2681 | -1.38 | 5.89e-05 | 0.0182 |
| A_09_P041151 | 40444 | Act79B | -1.35 | 7.66e-05 | 0.0205 |
| A_09_P037846 | 32128 | CG9360 | -1.34 | 4.56e-05 | 0.0161 |
| A_09_P008666 | 36955 | CG4802 | -1.29 | 4.45e-05 | 0.0159 |
| A_09_P046641 | 43828 | CaMKII | -1.28 | 9.71e-05 | 0.0225 |
| A_09_P123760 | 36927 | GstS1 | -1.25 | 0.000199 | 0.0295 |
| A_09_P075821 | 41994 | CG14882 | -1.25 | 0.000165 | 0.0273 |
| A_09_P028561 | 38243 | CG12024 | -1.25 | 0.000322 | 0.0401 |
| A_09_P050761 | 39293 | Mob2 | -1.24 | 6e-05 | 0.0183 |
| A_09_P028771 | 38323 | CG16985 | -1.23 | 0.000446 | 0.0482 |
| A_09_P009441 | 37217 | CG7461 | -1.2 | 0.000286 | 0.0368 |
| A_09_P041326 | 39041 | Argk | -1.19 | 2.66e-05 | 0.0123 |
| A_09_P008946 | 37054 | CG5770 | -1.17 | 0.000236 | 0.0326 |
| A_09_P073136 | 41167 | SpdS | -1.16 | 0.000385 | 0.0441 |
| A_09_P007091 | 36425 | CG17019 | -1.13 | 0.000466 | 0.0493 |
| A_09_P061066 | 43513 | CG7837 | -1.12 | 0.000114 | 0.0232 |
| A_09_P146045 | 36307 | Oda | -1.11 | 0.000386 | 0.0441 |
| A_09_P217395 | 39301 | CG7368 | -1.04 | 0.000429 | 0.0471 |
| A_09_P035926 | 40522 | CG12581 | -1.02 | 8.21e-05 | 0.0206 |
| A_09_P148450 | 40623 | CG2604 | -0.993 | 0.000471 | 0.0495 |
| A_09_P164245 | 39854 | CG4098 | 0.827 | 0.000349 | 0.0422 |
| A_09_P005901 | 36034 | CG15863 | 0.899 | 0.00045 | 0.0482 |
| A_09_P064946 | 34191 | CG17834 | 0.91 | 0.000464 | 0.0492 |
| A_09_P053486 | 38805 | CG8607 | 1.03 | 0.00044 | 0.048 |
| A_09_P016761 | 31491 | CG33080 | 1.04 | 0.000139 | 0.0253 |
| A_09_P064596 | 59212 | CG18854 | 1.09 | 0.000296 | 0.0377 |
| A_09_P057906 | 37595 | CG30196 | 1.1 | 0.000342 | 0.0416 |
| A_09_P079806 | 40713 | CG2082 | 1.1 | 0.000373 | 0.0432 |
| A_09_P037381 | 31989 | CG2124 | 1.1 | 0.000122 | 0.0244 |
| A_09_P050431 | 36001 | dap | 1.1 | 0.000164 | 0.0273 |
| A_09_P101040 | 3355150 | AGO3 | 1.13 | 0.000372 | 0.0432 |
| A_09_P076441 | 41643 | Lip3 | 1.14 | 0.000336 | 0.0411 |
| A_09_P115935 | 40324 | CG10584 | 1.15 | 0.000114 | 0.0232 |
| A_09_P024196 | 3355132 | CG14464 | 1.16 | 8.38e-05 | 0.0208 |

| | | | | | |
|---|---|---|---|---|---|
| A_09_P036041 | 40553 | Cont | 1.16 | 0.000371 | 0.0432 |
| A_09_P057671 | 246460 | CG30108 | 1.16 | 2.2e-05 | 0.0109 |
| A_09_P023066 | 35082 | CG15152 | 1.16 | 5.44e-05 | 0.0174 |
| A_09_P005601 | 35928 | CG11784 | 1.18 | 0.000363 | 0.0429 |
| A_09_P070756 | 42800 | CG10232 | 1.18 | 0.00014 | 0.0254 |
| A_09_P020106 | 33941 | Tsp | 1.24 | 0.000188 | 0.0287 |
| A_09_P131640 | 34282 | GlcAT-S | 1.26 | 0.000421 | 0.0464 |
| A_09_P069101 | 42294 | Cyp12a4 | 1.26 | 0.000403 | 0.0456 |
| A_09_P179200 | 3772557 | pncr012:2L | 1.28 | 0.000105 | 0.0232 |
| A_09_P067391 | 31512 | CG15766 | 1.28 | 0.000363 | 0.0429 |
| A_09_P034306 | 40035 | Sgf11 | 1.3 | 0.000175 | 0.0279 |
| A_09_P030951 | 40909 | alpha-Est1 | 1.33 | 0.00045 | 0.0482 |
| A_09_P044831 | 32498 | sog | 1.37 | 0.000136 | 0.0253 |
| A_09_P066201 | 35932 | CG8788 | 1.38 | 0.000145 | 0.0259 |
| A_09_P060431 | 261623 | CG31683 | 1.41 | 0.000124 | 0.0246 |
| A_09_P057496 | 246425 | CG30059 | 1.43 | 0.000421 | 0.0464 |
| A_09_P045401 | 41520 | Cyp9f2 | 1.45 | 0.000235 | 0.0326 |
| A_09_P024241 | 35498 | TpnC4 | 1.48 | 0.000113 | 0.0232 |
| A_09_P030091 | 34615 | Rh5 | 1.48 | 0.00011 | 0.0232 |
| A_09_P166370 | 3771992 | pncr016:2R | 1.51 | 2.13e-06 | 0.00344 |
| A_09_P012691 | 44155 | Sip1 | 1.57 | 0.000102 | 0.0231 |
| A_09_P030841 | 35939 | Rme-8 | 1.57 | 9.1e-05 | 0.0218 |
| A_09_P007936 | 36689 | CG8079 | 1.64 | 1.32e-05 | 0.00787 |
| A_09_P070466 | 42711 | Or94a | 1.67 | 2.06e-05 | 0.0107 |
| A_09_P004541 | 5740130 | CG34454 | 1.68 | 0.000159 | 0.0271 |
| A_09_P069096 | 42293 | Cyp12a5 | 1.69 | 0.000194 | 0.0291 |
| A_09_P025806 | 37341 | CG11159 | 1.69 | 0.000408 | 0.0458 |
| A_09_P058466 | 246614 | CG30438 | 1.71 | 0.00011 | 0.0232 |
| A_09_P032156 | 33119 | mst | 1.72 | 8.01e-06 | 0.00582 |
| A_09_P189040 | 39917 | rogdi | 1.73 | 0.000311 | 0.0393 |
| A_09_P059616 | 43177 | CG31324 | 1.81 | 1.78e-05 | 0.00995 |
| A_09_P115030 | 53542 | lectin-28C | 1.82 | 3.08e-05 | 0.0137 |
| A_09_P029071 | 38409 | dro5 | 1.88 | 0.00013 | 0.0253 |
| A_09_P062061 | 3355163 | Rab21 | 1.93 | 3.43e-05 | 0.0145 |
| A_09_P005791 | 36000 | CG10459 | 1.96 | 0.000475 | 0.0497 |
| A_09_P053926 | 38946 | CG17352 | 1.98 | 0.000247 | 0.0334 |
| A_09_P000061 | 3346224 | CG33510 | 2.01 | 1.91e-05 | 0.0101 |
| A_09_P192235 | 35417 | CG2201 | 2.02 | 6.46e-05 | 0.0188 |
| A_09_P180825 | 36399 | Obp49a | 2.03 | 0.000217 | 0.0309 |
| A_09_P063896 | 50442 | CG14933 | 2.04 | 7.95e-05 | 0.0205 |
| A_09_P002196 | 2768859 | CG33226 | 2.06 | 0.000132 | 0.0253 |
| A_09_P216565 | 37572 | CG6018 | 2.09 | 3.43e-06 | 0.00392 |
| A_09_P211320 | 50320 | dpr12 | 2.1 | 8.14e-05 | 0.0205 |
| A_09_P001201 | 3885601 | CG34054 | 2.17 | 0.000137 | 0.0253 |
| A_09_P035476 | 40381 | CG7529 | 2.17 | 0.000299 | 0.0381 |
| A_09_P053601 | 38840 | Cyp316a1 | 2.25 | 1.72e-06 | 0.00344 |
| A_09_P194045 | 32977 | CG14224 | 2.25 | 0.000133 | 0.0253 |
| A_09_P137955 | 33863 | stai | 2.26 | 2.41e-05 | 0.0113 |
| A_09_P077386 | 31102 | CG14780 | 2.3 | 6.2e-05 | 0.0184 |
| A_09_P044706 | 31617 | shf | 2.39 | 3.77e-05 | 0.0152 |
| A_09_P008366 | 36840 | CG4409 | 2.39 | 0.000175 | 0.0279 |
| A_09_P072971 | 41116 | CG18473 | 2.4 | 6.43e-05 | 0.0188 |
| A_09_P025156 | 35821 | Cyp4ad1 | 2.42 | 1.02e-05 | 0.00661 |
| A_09_P042701 | 44008 | Hex-C | 2.52 | 6.55e-05 | 0.0188 |
| A_09_P106615 | 40675 | CRMP | 2.53 | 2.13e-06 | 0.00344 |
| A_09_P032616 | 33636 | Atet | 2.53 | 0.000101 | 0.023 |
| A_09_P025186 | 35837 | Cyp6a13 | 2.54 | 1.88e-05 | 0.0101 |

| | | | | | |
|---|---|---|---|---|---|
| A_09_P104310 | 44038 | Hf | 2.56 | 2.01e-06 | 0.00344 |
| A_09_P014181 | 319045 | CG31955 | 2.64 | 0.000235 | 0.0326 |
| A_09_P063466 | 50207 | CG15065 | 2.64 | 0.000154 | 0.0268 |
| A_09_P077236 | 31086 | CG3056 | 2.73 | 0.000125 | 0.0246 |
| A_09_P009261 | 37165 | Mctp | 2.77 | 5.49e-08 | 0.000798 |
| A_09_P032981 | 41313 | Sodh-2 | 2.78 | 4.44e-05 | 0.0159 |
| A_09_P029611 | 35767 | Odc2 | 2.92 | 8.14e-05 | 0.0205 |
| A_09_P137330 | 32858 | Cyp18a1 | 2.93 | 0.000312 | 0.0393 |
| A_09_P035096 | 40263 | CG11796 | 2.97 | 3.3e-06 | 0.00388 |
| A_09_P045756 | 34761 | Tehao | 3.04 | 7.05e-06 | 0.00559 |
| A_09_P011886 | 48336 | GstD3 | 3.12 | 3.44e-08 | 0.000798 |
| A_09_P020721 | 34131 | CG8086 | 3.16 | 5.68e-06 | 0.00496 |
| A_09_P019531 | 33756 | Cyp4ac3 | 3.24 | 5.27e-06 | 0.00472 |
| A_09_P044531 | 34570 | sala | 3.51 | 8.62e-07 | 0.00273 |
| A_09_P016781 | 42654 | CG33093 | 3.6 | 8.33e-07 | 0.00273 |
| A_09_P036596 | 40728 | CG1213 | 3.63 | 2.3e-05 | 0.011 |
| A_09_P054741 | 39225 | Cpr67Fb | 3.65 | 7.75e-05 | 0.0205 |
| A_09_P193360 | 34144 | CG14275 | 3.76 | 3.23e-06 | 0.00388 |
| A_09_P007281 | 36486 | IM10 | 4.03 | 0.000103 | 0.0232 |
| A_09_P177575 | 36636 | AttA | 4.18 | 0.000178 | 0.0282 |
| A_09_P055541 | 39466 | CG12520 | 4.19 | 1.73e-06 | 0.00344 |
| A_09_P040846 | 33047 | CG1702 | 4.75 | 9.96e-05 | 0.0229 |
| A_09_P031136 | 45556 | Cyp6a17 | 5.3 | 1.87e-05 | 0.0101 |
| A_09_P018246 | 33312 | CG42329 | 5.36 | 1.56e-06 | 0.00344 |
| A_09_P007261 | 36481 | CG10814 | 6.71 | 4.33e-05 | 0.0159 |
