# Supplementary material for: Mondo/ChREBP-Mlx-Regulated Transcriptional Network Is Essential for Dietary Sugar Tolerance in Drosophila
Source: PLoS Genet. 2013 Apr 4;9(4):e1003438. doi: 10.1371/journal.pgen.1003438 (PMC3616910; doi:10.1371/journal.pgen.1003438)
Supplement: Table S2 — Systematic in vivo RNAi screen of 103 Mlx candidate target genes for sugar sensitivity. Embryonic lethal: embryonic lethality on both diets, larval lethal: larval lethality on both diets, pupal lethal/no phenotype: equal pupation on both diets leading to pupal lethality, no phenotype: equal adult emergence on both diets, sugar sensitive: reduced pupation or adult emergence on high sugar (20% yeast-15% sucrose) diet, N/D: no data. GD and KK-lines from Vienna Drosophila RNAi Center. NIG-line from NIG-Fly Stock Center. *) Early larval lethality with yeast+15% sucrose diet, late larval lethality with yeast diet. (PDF) [file pgen.1003438.s008.pdf]

Supplemental table 2.

CG number	gene	logFC	adj.P. Val	human ortholog (from Flybase)	tub-GAL4> phenotype in yeast vs. yeast+15% sucrose	RNAi lines tested
CG4427	cabut	-4.024	0.004	Kruppel-like factor 12	sugar sensitive *)	4427 R-1 NIG
CG3368	CG3368	-3.934	0.003	-	no phenotype	26177 GD
CG7231	CG7231	-3.895	0.001	family with sequence similarity 151, member B	N/D	
CG1944	Cyp4p2	-3.871	0.004	-	no phenotype	1498 GD
CG1600	CG1600	-3.75	0.016	-	no phenotype	108325 KK
CG17642	mitochondrial ribosomal protein L48	-3.7	0.004	mitochondrial ribosomal protein L48	larval lethal	107308 KK
CG3348	CG3348	-3.524	0.003	-	no phenotype	8690 GD
CG2043	Larval cuticle protein 3	-3.28	0.096	-	larval lethal	107682 KK
CG3609	CG3609	-3.271	0.01	dihydrodiol dehydrogenase	larval lethal	108742 KK
CG12068	shroud	-3.051	0.023	-	larval lethal	50112 GD, 16388 GD
CG1774	CG1774	-3.05	0.266	-	no phenotype	101033 KK
CG34301	CG34301	-2.891	0.011	-	embryonic lethal	105343 KK
CG15347	CG15347	-2.88	0.01	-	no phenotype	107479 KK
CG3705	astray	-2.80	0.118	phosphoserine phosphatase	pupal lethal/no phenotype	110661 KK
CG6426	CG6426	-2.779	0.017	-	N/D	
CG34141	CG34141	-2.714	0.004	-	N/D	
CG2044	Larval cuticle protein 4	-2.70	0.308	-	pupal lethal/no phenotype	105025 KK
CG15257	Translocase inner membrane 17	-2.697	0.018	-	no phenotype	3837 GD
CG6074	CG6074	-2.66	0.073	-	pupal lethal/no phenotype	31138 GD
CG8936	Arpc3B	-2.65	0.043	-	N/D	
CG8256	Glycerophosphate oxidase-1	-2.61	0.296	glycerol-3-phosphate dehydrogenase 2 (mitochondrial)	pupal lethal/no phenotype	110608 KK
CG17322	CG17322	-2.556	0.005	-	embryonic lethal	110057 KK
CG32019	bent	-2.528	0.012	-	N/D	
CG15117	CG15117	-2.53	0.495	glucuronidase, beta	pupal lethal/no phenotype	108570 KK
CG8628	CG8628	-2.46	0.608	-	larval lethal	110103 KK
CG9042	Glycerol 3 phosphate dehydrogenase	-2.46	0.007	glycerol-3-phosphate dehydrogenase 1	larval lethal	105359 KK, 29015 GD

CG9432	lethal (2) 01289	-2.44	0.022	-	embryonic lethal	107350 KK
CG34397	Rgk3	-2.426	0.016	RAS (RAD and GEM)-like GTP-binding 1	no phenotype	106428 KK
CG32490	complexin	-2.40	0.057	-	no phenotype	21477 GD
CG8034	CG8034	-2.326	0.011	-	larval lethal	106660 KK
CG7675	CG7675	-2.324	0.023	-	larval lethal	107833 KK
CG10079	Epidermal growth factor receptor	-2.31	0.151	v-erb-b2 erythroblastic leukemia viral oncogene homolog 3 (avian)	pupal lethal/no phenotype	107130 KK
CG11129	Yolk protein 3	-2.29	0.317	-	pupal lethal/no phenotype	46787 GD
CG5773	CG5773	-2.266	0.021	-	no phenotype	106345 KK
CG8709	CG8709	-2.183	0.048	lipin 3	larval lethal	36007 GD
CG4563	CG4563	-2.12	0.649	-	no phenotype	101849 KK
CG13741	CG13741	-2.12	0.021	-	N/D	
CG18255	Stretchin-Mlck	-2.114	0.03	-	N/D	
CG9930	E5	-2.09	0.025	-	no phenotype	47794 GD
CG7478	Actin 79B	-2.077	0.025	-	N/D	
CG2139	aralar1	-2.044	0.017	solute carrier family 25 (mitochondrial carrier, Aralar), member 12	N/D	
CG11236	CG11236	-2.043	0.016	-	no phenotype	38460 GD
CG17608	fu12	-1.99	0.068	-	no phenotype	108540 KK
CG10699	Lim3	-1.987	0.006	LIM homeobox 3	pupal lethal/no phenotype	105746 KK
CG5991	CG5991	-1.974	0.025	phosphatidylserine decarboxylase	larval lethal	109033 KK
CG12946	Whamy	-1.97	0.046	-	N/D	
CG34288	CG34288	-1.966	0.031	-	N/D	
CG6933	CG6933	-1.96	0.737	-	no phenotype	104107 KK
CG5170	Dodeca-satellite-binding protein 1	-1.955	0.004	high density lipoprotein binding protein	no phenotype	106047 KK
CG6357	CG6357	-1.939	0.019	-	N/D	
CG5887	desat1	-1.907	0.016	stearoyl-CoA desaturase (delta-9-desaturase)	embryonic lethal	104350 KK
CG30463	CG30463	-1.883	0.019	-	pupal lethal/no phenotype	4923 GD
CG11140	Aldehyde dehydrogenase type III	-1.843	0.008	aldehyde dehydrogenase 3 family, member B1	sugar sensitive	107110 KK
CG5725	fumble	-1.823	0.027	pantothenate kinase 1	no phenotype	101437 KK
CG34418	still life	-1.821	0.015	-	no phenotype	106832 KK
CG12665	Odorant-binding protein 8a	-1.81	0.161	-	no phenotype	47798 GD
CG8665	CG8665	-1.79	0.155	aldehyde dehydrogenase 1 family, member L1	no phenotype	36000 GD

CG15553	CG15553	-1.79	0.343	-	pupal lethal/no phenotype	104514 KK
CG15361	Neuropeptide-like precursor 4	-1.79	0.411	-	no phenotype	104662 KK
CG7384	CG7384	-1.78	0.021	-	N/D	
CG10242	Cyp6a23	-1.75	0.246	-	no phenotype	100679 KK
CG2718	Glutamine synthetase 1	-1.728	0.034	-	no phenotype	40174 GD
CG13277	LSm7	-1.727	0.003	-	larval lethal	23863 GD
CG3879	Multi drug resistance 49	-1.72	0.135	-	no phenotype	108327 KK
CG2985	Yolk protein 1	-1.708	0.043	-	larval lethal	106735 KK
CG5436	Heat shock protein 68	-1.703	0.021	-	pupal lethal/no phenotype	107356 KK
CG1242	Heat shock protein 83	-1.70	0.334	heat shock protein 90kDa alpha (cytosolic), class A member 1	larval lethal	108568 KK
CG7533	charybde	-1.69	0.099	-	larval lethal	105757 KK
CG9447	CG9447	-1.68	0.082	-	no phenotype	7342 GD
CG14723	Histamine-gated chloride channel subunit 1	-1.66	0.652	-	pupal lethal/no phenotype	104966 KK
CG1410	waclaw	-1.658	0.036	GUF1 GTPase homolog (S. cerevisiae)	no phenotype	50569 GD
CG17633	CG17633	-1.65	0.04	-	no phenotype	108462 KK
CG14222	CG14222	-1.652	0.042	N(alpha)-acetyltransferase 20, NatB catalytic subunit	pupal lethal/no phenotype	109664 KK
CG12116	CG12116	-1.65	0.082	-	no phenotype	6498 GD
CG11218	Odorant-binding protein 56d	-1.64	0.547	-	no phenotype	100671 KK
CG17018	CG17018	-1.64	0.191	-	no phenotype	106964 KK
CG31623	defective transmitter release	-1.64	0.279	leucine rich repeat containing 50	no phenotype	103600 KK
CG31100	CG31100	-1.64	0.376	-	no phenotype	42630 GD
CG15120	CG15120	-1.628	0.043	PARK2 co-regulated	pupal lethal/no phenotype	107946 KK
CG41265	lethal (2) 41Ab	-1.622	0.046	-	N/D	
CG7266	Ecdysone-induced protein 28/29kD	-1.617	0.016	methionine sulfoxide reductase A	no phenotype	104465 KK
CG4373	Cyp6d2	-1.61	0.15	-	no phenotype	101829 KK
CG6186	Transferrin 1	-1.60	0.06	-	pupal lethal/no phenotype	106479 KK
CG4774	CG4774	-1.598	0.015	cardiolipin synthase 1	larval lethal	108392 KK
CG17270	CG17270	-1.597	0.029	-	pupal lethal/no phenotype	106420 KK
CG13188	CG13188	-1.577	0.029	-	larval lethal	102370 KK
CG18730	Amylase proximal	-1.57	0.498	amylase, alpha 2B (pancreatic)	no phenotype	50098 GD
CG31522	CG31522	-1.566	0.025	-	larval lethal	37329 GD
CG31620	Gustatory receptor 39b	-1.563	0.016	-	no phenotype	33215 GD
CG4830	CG4830	-1.56	0.674	-	no phenotype	21633 GD
CG10142	Ance-5	-1.553	0.03	-	no phenotype	15246 GD

CG8896	18 wheeler	-1.54	0.098	-	no phenotype	44387 GD
CG33968	drop dead	-1.54	0.029	-	no phenotype	103032 KK
CG11094	doublesex	-1.53	0.781	DMRT-like family B with proline-rich C-terminal, 1	larval lethal	110306 KK
CG4408	CG4408	-1.526	0.045	-	no phenotype	15235 GD
CG5804	CG5804	-1.53	0.439	-	no phenotype	23587 GD
CG17633	CG17633	-1.52	0.289	-	no phenotype	108462 KK
CG1641	sisterless A	-1.52	0.438	-	no phenotype	11797 GD
CG18557	CG18557	-1.44	0.033	-	no phenotype	107357 KK
CG13917	CG13917	-1.424	0.011	-	larval lethal	32082 GD
CG10391	Cyp310a1	-1.424	0.025	-	no phenotype	100318 KK
CG9542	CG9542	-1.408	0.025	arylformamidase	embryonic lethal	109675 KK
CG2681	CG2681	-1.376	0.018	-	no phenotype	110791 KK
CG9360	CG9360	-1.341	0.016	dehydrogenase/reductase (SDR family) member 11	no phenotype	13189 GD
CG4802	CG4802	-1.293	0.016	methylthioadenosine phosphorylase	no phenotype	108304 KK
CG18069	Calcium/calmodulin-dependent protein kinase II	-1.282	0.023	calcium/calmodulin-dependent protein kinase II delta	no phenotype	100265 KK
CG1725	discs large 1	-1.281	0.021	discs, large homolog 1 (Drosophila)	no phenotype	109274 KK
CG14882	CG14882	-1.266	0.033	-	larval lethal	103984 KK
CG8938	Glutathione S transferase S1	-1.252	0.029	hematopoietic prostaglandin D synthase	larval lethal	50140 GD
CG12024	CG12024	-1.249	0.04	CUE domain containing 1	no phenotype	20143 GD
CG16985	CG16985	-1.232	0.048	acyl-CoA thioesterase 13	no phenotype	107909 KK
CG7461	CG7461	-1.212	0.043	acyl-CoA dehydrogenase, very long chain	no phenotype	110220 KK
CG32031	Arginine kinase	-1.204	0.03	creatine kinase, brain	larval lethal	34036 GD
CG5770	CG5770	-1.174	0.033	-	N/D	
CG8327	Spermidine Synthase	-1.173	0.05	spermidine synthase	no phenotype	10877 KK
CG17019	CG17019	-1.131	0.049	ring finger protein 34	no phenotype	48749 GD
CG7837	CG7837	-1.123	0.023	-	no phenotype	109784 KK
CG16747	Ornithine decarboxylase antizyme	-1.106	0.044	ornithine decarboxylase antizyme 3	N/D	
CG7368	CG7368	-1.038	0.047	-	pupal lethal/no phenotype	109515 KK
