# Supplementary material for: Mondo/ChREBP-Mlx-Regulated Transcriptional Network Is Essential for Dietary Sugar Tolerance in Drosophila
Source: PLoS Genet. 2013 Apr 4;9(4):e1003438. doi: 10.1371/journal.pgen.1003438 (PMC3616910; doi:10.1371/journal.pgen.1003438)
Supplement: Table S3 — Primer sequences used in quantitative RT-PCR. (PDF) [file pgen.1003438.s009.pdf]

Supplemental table 3.

Gene	***Forward***	***Reverse***
rp49	AGGGTATCGACAACAGAGTG	CACCAGGAACTTCTTGAATC
actin	CCGTACCACAGGTATCGTGTTG	GTCGGTTAAATCGCGACCG
cabut	ATGCCTTCTCGCTCTCATGT	TCCTGGAAAGAAGTGGCATC
Aldh-III	GATCAAGCCCAGCGAGATT	CGCAGACAACTGGATAGCAA
mondo	GCGGCGTTACAACATAAAGA	CTCCATGCGCAAAGCTTCAA
mlx	GCCAAGTTTCAAGTGTTCCAG	CTCCAGCCAGGGGATAATG
Fas	CTCCACCATCGAGGAGTTCA	CTTGAGCTTGCCAATCCTGT
ACC	GGCTATGCTGCGCTTAACA	GCCTCTGTTTTGTGGGTGAC
desat1	GCGCGCCGATCCAATCCTCA	GGTGGCCACAAACCAGGCGT
Gs1	TTGTCCTGTGCGACACCTAC	ATTCTTGCTCAATGCCGAAC
Gpdh	GCAAGCTGTCCACCTTCTTC	CAGAAGTCACAAACGCCTCA
Gpo-1	AGATGGGACACTCGGTCAAC	TGATGGAAACGTAGCCCTTC
PFK2	AGAGCGAGTACAACCTGAGC	TAGCGCATTGGCATACTGGT
sro	TGCTGGAGTAATGTGCTTCG	TCATGTGTCAGCCTCATGGT
